# Metabolic alterations in pea leaves during arbuscular mycorrhiza development

**DOI:** 10.7717/peerj.7495

**Published:** 2019-08-23

**Authors:** Oksana Y. Shtark, Roman K. Puzanskiy, Galina S. Avdeeva, Andrey P. Yurkov, Galina N. Smolikova, Vladislav V. Yemelyanov, Marina S. Kliukova, Alexey L. Shavarda, Anastasiia A. Kirpichnikova, Aleksandr I. Zhernakov, Alexey M. Afonin, Igor A. Tikhonovich, Vladimir A. Zhukov, Maria F. Shishova

**Affiliations:** 1 Department of Biotechnology, All-Russia Research Institute for Agricultural Microbiology, Pushkin, St. Petersburg, Russia; 2 Faculty of Biology, St. Petersburg State University, St. Petersburg, Russia; 3 Laboratory of Dynamics of Arctic Vegetation, Komarov Botanical Institute of the Russian Academy of Sciences, St. Petersburg, Russia; 4 Center for Molecular and Cell Technologies, St. Petersburg State University, St. Petersburg, Russia

**Keywords:** *Pisum sativum*, Arbuscular mycorrhiza, Plant growth and physiological state, Leaf, Metabolic profile

## Abstract

Arbuscular mycorrhiza (AM) is known to be a mutually beneficial plant-fungal symbiosis; however, the effect of mycorrhization is heavily dependent on multiple biotic and abiotic factors. Therefore, for the proper employment of such plant-fungal symbiotic systems in agriculture, a detailed understanding of the molecular basis of the plant developmental response to mycorrhization is needed. The aim of this work was to uncover the physiological and metabolic alterations in pea (*Pisum sativum* L.) leaves associated with mycorrhization at key plant developmental stages. Plants of pea cv. Finale were grown in constant environmental conditions under phosphate deficiency. The plants were analyzed at six distinct time points, which corresponded to certain developmental stages of the pea: I: 7 days post inoculation (DPI) when the second leaf is fully unfolded with one pair of leaflets and a simple tendril; II: 21 DPI at first leaf with two pairs of leaflets and a complex tendril; III: 32 DPI when the floral bud is enclosed; IV: 42 DPI at the first open flower; V: 56 DPI when the pod is filled with green seeds; and VI: 90–110 DPI at the dry harvest stage. Inoculation with *Rhizophagus irregularis* had no effect on the fresh or dry shoot weight, the leaf photochemical activity, accumulation of chlorophyll *a*, *b* or carotenoids. However, at stage III (corresponding to the most active phase of mycorrhiza development), the number of internodes between cotyledons and the youngest completely developed leaf was lower in the inoculated plants than in those without inoculation. Moreover, inoculation extended the vegetation period of the host plants, and resulted in increase of the average dry weight per seed at stage VI. The leaf metabolome, as analyzed with GC-MS, included about three hundred distinct metabolites and showed a strong correlation with plant age, and, to a lesser extent, was influenced by mycorrhization. Metabolic shifts influenced the levels of sugars, amino acids and other intermediates of nitrogen and phosphorus metabolism. The use of unsupervised dimension reduction methods showed that (i) at stage II, the metabolite spectra of inoculated plants were similar to those of the control, and (ii) at stages IV and V, the leaf metabolic profiles of inoculated plants shifted towards the profiles of the control plants at earlier developmental stages. At stage IV the inoculated plants exhibited a higher level of metabolism of nitrogen, organic acids, and lipophilic compounds in comparison to control plants. Thus, mycorrhization led to the retardation of plant development, which was also associated with higher seed biomass accumulation in plants with an extended vegetation period. The symbiotic crosstalk between host plant and AM fungi leads to alterations in several biochemical pathways the details of which need to be elucidated in further studies.

## Introduction

The vast majority of land plants form arbuscular mycorrhizae (AM), symbioses with obligatorily biotrophic Glomeromycota fungi. Arbuscular-mycorrhizal fungi (AMF) feed on photosynthesis products and utilize a considerable proportion of the assimilated carbon. They form intraradical colonies with branched intracellular structures, the arbuscules. The arbuscules are surrounded by a membrane which supports bidirectional transport of nutrients: inorganic phosphate, ammonium, and other mineral ions flow from the fungal cell to the plant cell, and carbohydrates are transferred in the opposite direction (Smith & Read, 2008; Kaschuk et al., 2009; Gutjahr & Parniske, 2013; Tisserant et al., 2012; Manck-Götzenberger & Requena, 2016). Recently it has also been shown that the growth and development of AMF depends on lipid transfer from the host plant (Luginbuehl et al., 2017). The AM is generally considered to be a mutually beneficial symbiosis that facilitates plant nutrition and increases plant tolerance to biotic and abiotic stresses (Siddiqui, Akhtar & Futai, 2008; Solaiman, Abbott & Varma, 2014). At the same time, it has been increasingly recognized that interactions may follow a continuum from mutualistic to parasitic. The effect of mycorrhization on a plant depends on soil ecology, light conditions, plant and fungal species and their mutual cross-adaptation (Klironomos, 2003; Xavier & Germida, 2003; Smith & Read, 2008; Schweiger et al., 2014; Konvalinková & Jansa, 2016). Despite the negative growth response to mycorrhization under certain conditions, plants with formed AM might still have better fitness than the non-mycorrhizal control plants because of their better nutrition (Koide, 2010).

The pea (*Pisum sativum* L.) is an important legume crop, which forms both AM and nitrogen-fixing root nodules with rhizobia (Vance, 2008). Combined inoculation with both AMF and rhizobia can lead to a 3-fold and greater increase in plant biomass and seed mass in many pea genotypes compared to mono-inoculation with rhizobia (Jacobi et al., 1999). A separate work showed combined inoculation to lead to an increase in seed protein content in most tested lines (Shtark et al., 2006). However, *P. sativum* had a relatively low growth response to mono-inoculation with AMF compared to *Medicago varia*, *Secale cereale* or *Hordeum vulgare*, as was demonstrated in the geographical network experiments of the All-Russia Research Institute for Agricultural Microbiology (St. Petersburg) (Yurkov et al., 2017). Furthermore, many authors have described the complete absence of an increase of pea growth parameters under conditions of mono-inoculation with AMF (Rivera-Becerril et al., 2002; Xavier & Germida, 2003; Borisov et al., 2004b; Desalegn et al., 2016; Zhukov et al., 2017). Even pea genotypes having the highest growth response to double inoculation may not show a positive response to mono-inoculation with AMF (Borisov et al., 2004b). All this data indicate the necessity of a more detailed understanding of the molecular and genetic bases of AMF interactions with the host plant. These aspects of the AM formation *per se* are particularly well studied for *Medicago truncatula* and *Lotus japonicus* (Gutjahr & Parniske, 2013; Gobbato, 2015; Pimprikar & Gutjahr, 2018). Information for *Pisum sativum* (Borisov et al., 2004a; Kuznetsova et al., 2010; Zhukov et al., 2016; Shtark et al., 2016; Leppyanen et al., 2017) is scarce and its physiological and biochemical aspects need further study.

Metabolomics is a powerful tool for investigating a plant’s physiological/biochemical status varied under different environmental conditions (Iriti & Vitalini, 2018; Peters et al., 2018). Recent studies have revealed the species-specificity of leaf metabolic responses to AM, implying that various metabolites can be affected. Such mycorrhiza-mediated changes in the chemical composition of leaf tissues can impart phytoprotection against different abiotic stresses (Schweiger et al., 2014; Schweiger & Mueller, 2015). In *P. sativum* the studies were mainly focused on the seed metabolome, as was expected for a pulse crop, and a lot of attention was also paid to the effects of different stress conditions (Charlton et al., 2008; Vigeolas et al., 2008; Cechová et al., 2017; Hradilová et al., 2017; Sistani et al., 2017; Woźniak et al., 2017; Ellis et al., 2018; Tasho, Shin & Cho, 2018). However, Desalegn et al. (2016) recently reported on leaf metabolome changes driven by inoculation with AMF and rhizobia of healthy pea plants and those infected with a pathogenic fungus *Didymella pinodes*.

The aim of the present study was to analyze the effect of inoculation with AMF *Rhizophagus irregularis* on growth and the physiological and biochemical state of pea plants. Detailed analysis of the leaf metabolome using gas chromatography-mass spectrometry (GC-MS), along with investigation of leaf photochemical activity and pigment content, was performed to assess the changes caused in *P. sativum* leaves by mycorrhization at the key stages of plant development. Despite the fact that plants did not show a strong growth response to the inoculation, and their photosynthetic activity was not affected by mycorrhization, this study revealed significant metabolic alterations occurring in pea leaves during the development of AM symbiosis. These alterations were associated with a prolongation of the vegetation period and an increase in the seed biomass of inoculated plants.

## Materials & Methods

### Plant and fungal material

The low, determinate pea (*Pisum sativum* L.) cv. Finale (Cebeco, Rotterdam, The Netherlands) with dark green leaves, white flowers and round green seeds (Engvild, 1987) was used to study AM mediated metabolic alterations in leaves. This cultivar was used because of its stable yields and wide adaptation (Engvild, 1987).

The fungal isolate *Rhizophagus irregularis* BEG144 used in the study was previously characterized as forming highly effective AM symbioses with many agricultural crops (Muromtsev, Marshunova & Jacobi, 1989). The isolate was initially provided by the International Bank for the Glomeromycota (Dijon, France) and has been maintained in *Plecthrantus australis* pot cultures. To produce the inoculum of the fungus for this experiment, mycorrhizal *Sorghum* sp. plants were grown in pot cultures (see ‘Fungal inoculum preparation’).

### Plant growth conditions

All plants used in this work were grown in pots with a growth substrate consisting of sterile soil and quartz sand mixture (1:2 v/v), supplemented with 1 g L^−1^ Ca_3_PO_4_ as a source of phosphate. A loamy sandy soddy-podzolic soil obtained from Gatchinsky district, Leningrad Oblast, Russia, with the following characteristics was used: pH (KCl) 4.8; 3.6% organic matter; 35 mg kg^−1^ available K_2_O (extraction with 0.2 N HCl); 33 mg kg^−1^ available P_2_O_5_ (extraction with 0.2 N HCl); 28,7 mg-equivalent kg^−1^ hydrolytic acidity; 98 mg-equivalent kg^−1^ base exchange materials. CaCO_3_ (1.44 g kg^−1^) was added to the soil to correct pH. Pots with the growth substrate were autoclaved twice with a two-day interval for 60 min at 134 °C and 0.22 MPa to remove soil microbiota and kept for a month to eliminate volatile toxic compounds. Plants were grown in a constant environment chamber (model VB 1514; Vštsch, Hanau, Germany) at 16/8 h and 24/22C day/night regime, 75% relative humidity, and around 10,000 lux illumination. The pot cultures were fertilized once a week with 0.5× modified Hoagland’s solution without phosphate (Shtark et al., 2016) (0.15 L L^−1^ of the growth substrate), and watered as needed.

### Fungal inoculum preparation

*Sorghum* seeds were surface-disinfected as follows: 1 min in 96% ethanol, a rinse with sterile water, 10 min in 0.15% KMnO_4_ aqueous solution and a thorough rinse with sterile water. Disinfected *Sorghum* seeds were sown in the growth substrate (see ‘Plant growth conditions’), supplemented with 1g L^−1^ fresh and washed *P. australis* mycorrhizal roots. After 120 days of cultivation under conditions described in ‘Plant growth conditions’, *Sorghum* root systems were extracted from the growth substrate, cut into one cm pieces, and dried at room temperature and then mixed again with the growth substrate they were extracted from at a 1:1 ratio (v/v).

### Experimental design and collection of plant material

Pea seeds were surface-disinfected as follows: 1 min in 96% ethanol, a rinse with sterile water, 8 min in a 5% NaClO aqueous solution, and a thorough rinse with sterile water. Disinfected pea seeds were germinated on sterile humid vermiculite in Petri dishes for 3 days at 27 °C in the dark. Two pea seedlings of equal size were planted into a 300-ml ceramic flower pot with the growth substrate described in ‘Plant growth conditions’. Half of the pots were supplemented with 15 g L^−1^ *R. irregularis* inoculum before planting; the other half was left as control.

The plants were analyzed at six points in time, which corresponded to specific developmental stages for the growth of this species. I: 7 days post inoculation, DPI (Vegetative stage, second leaf fully unfolded with one pair of leaflets, simple tendril); II: 21 DPI (Vegetative stage, first leaf with two pairs of leaflets, complex tendril); III: 32 DPI (Reproductive stage, an enclosed floral bud); IV: 42 DPI (Reproductive stage, first open flower); V: 56 DPI (Reproductive stage, pod fill. Green seeds fill the pod cavity); and VI: 90–110 DPI (Senescence stage, dry harvest stage. All pods dry and brown, seed dry). The developmental stages were selected in accordance to Knott (1987) with the following difference: all the internodes were counted, including those adjoining the two small scale leaves, resulting in two additional nodes.

At stages I–V, ten or more plants per treatment were taken at random at each stage. The plants were removed from the soil and their root systems thoroughly washed. The total fresh weight, the fresh weight of the aerial part of the plants and the number of internodes were determined. After measuring the growth parameters, the youngest fully-developed leaf from each analyzed plant was cut off. Leaves from three to five plants were allocated to a single biological replicate, weighed and snap-frozen in liquid nitrogen in two mL Eppendorf Safe-Lock tube, and then stored at −80 °C. At least three biological replicates for each time point were collected for pigment and metabolome analysis. For arbuscular mycorrhiza analysis, fragments of lateral roots were collected individually from each plant in two mL Eppendorf tubes and were stored at –20 °C. At stage VI, the aerial parts of all remaining plants were collected and their dry weight was measured after drying at room temperature for three months.

### Analysis of mycorrhization

Sheaffer Black Ink staining was performed according to Vierheilig et al. (1998) to visualize fungal structures in the root samples. Roots were washed once with distilled water and covered in glycerol; root fragments totaling a length of 30 cm for each plant (*n* = 10) were mounted on glass slides. The AM development was examined using Axiovert 35 light microscope (Zeiss/Opton, Germany) and quantitatively assessed according to Trouvelot, Kough & Gianinazzi-Pearson (1986) by the following parameters: *M%* = intensity of intraradical mycelium development (reflects the proportion of the root length colonized by the fungus), and *a%* = arbuscule abundance in mycorrhizal root fragments (characterizes the functional state of the fungus). For statistical analysis, the parameters were subjected to arcsine transformation to normalize the data (Little & Hills, 1978).

### Leaf photochemical activity and pigment content analyses

Chlorophyll *a* fluorescence analysis was conducted at stages I–V on the day before the plant material sampling (6, 20, 31, 41, and 55 DPI). The photochemical activity of one of the leaflets in the first pair of the youngest fully formed leaf (Fig. S1) was measured. Plants (for stage V *n* = 5, for the rest stages *n* = 3) in pots were placed in a light-tight chamber and pre-adapted to darkness for 15 min before the measurements. The kinetics of the chlorophyll *a* fluorescence induction was acquired at room temperature by pulse amplitude modulation (PAM) fluorometric analysis using a portable chlorophyll fluorometer PAM-2500 (Heinz Walz GmbH, Effeltrich, Germany). To secure the leaflets, a 2030-B clamp equipped with a quantum and temperature sensor was used. The quantitative fluorescent parameters and related calculated factors were derived using the PAMWin-3 Software and Instruction manual for PAM-2500 (Heinz Walz, https://www.walz.com). Using the original data, the following values were obtained: *F*_*v*_∕*F*_*m*_, the maximum PSII photochemical efficiency in the darkness-adapted state (Kitajima & Butler, 1975); *Y(II)*, the effective quantum yield of photochemical energy conversion in PSII (Genty, Briantais & Baker, 1989); *qP*, the coefficient of photochemical quenching of chlorophyll fluorescence (Schreiber, Schliwa & Bilger, 1986); *qN*, the coefficient of non-photochemical quenching of chlorophyll fluorescence (Schreiber, Schliwa & Bilger, 1986). The maximum electron transport rate (*ETRmax*) at light saturation and minimum saturating irradiance (*Ik*) were also calculated.

The leaflet area was calculated using Fovea Pro v. 4.0 for Adobe Photoshop (Reindeer Graphics, http://reindeergraphics.com/products.html).

Leaf pigment analysis was conducted at stages I–V (for stage V *n* = 4, for the remaining stages *n* = 3). The leaf samples (up to 0.02 g) were ground three times for 2 min in two mL microtubes with 3 metal balls three mm in diameter in liquid nitrogen by using a Tissue Lyser LT (Qiagen, Hilden, Germany) bead mill at the 50 hits s^−1^ frequency. The pigments were quantitatively extracted with methanol as described by Smolikova et al. (2017). The absorption spectra of the extracts were acquired at 470.0, 652.4, and 665.2 nm in quartz cuvettes with one cm light path (Reachim, St. Petersburg, Russia) by using a UV/Vis spectrophotometer Spekol 1300 (Analytik Jena AG, Jena, Germany). The chlorophyll and carotenoid contents were calculated as recommended by Lichtenthaler (1987) and Lichtenthaler & Buschmann (2001) and normalized to fresh weights.

### Metabolome analysis

Leaves were sampled at stages II, IV and V. The samples (0.1–0.2 g) were ground as described by Puzanskiy et al. (2018) and subjected to a single-stage extraction with two mL methanol: chloroform: water (5:2:1) mixture. Tissue debris was removed by centrifugation at 12,000 g for 10 min at −5 °C. The supernatant was collected and evaporated in a vacuum evaporator (Eppendorf, Germany). The dried material was dissolved in pyridine with the internal tricosane standard (nC23). The samples were then supplied with the silylating agent BSTFA: TMCS 99:1 (Sigma-Aldrich) and derivatizated at 90 °C for 20 min (Puzanskiy et al., 2015; Puzanskiy et al., 2018).

GC-MS analysis was performed at Agilent 5860 chromatograph using Agilent ChemStation E.02.02.1431 software (Agilent Technologies, Santa Clara, CA, USA). Separation was performed on a J&W HP-5ms capillary column 30 m long 0.25 mm in diameter, stationary phase film (95% dimethylpolyoxane, 5% diphenyl), thickness 0.1 µm. The helium gas flow rate was one mL min^−1^. Inlet temperature was 250 °C at splitless mode. The temperature conditions of the column thermostat were: an initial temperature of 70 °C, increased by 5 °C per min up to 320 °C. The peaks were recorded by an Agilent 5975S mass selective detector (Agilent Technologies, Santa Clara, CA, USA). Electron impact ionization was performed at 70 V and an ion source temperature of 230 °C.

The analysis of the GC-MS data was processed using the PARADISe program (Department of Food Science Faculty of Science, University of Copenhagen, Denmark, http://www.models.life.ku.dk/paradise) coupled with NIST MS Search (National Institute of Standards and Technology (NIST), USA). In addition, the AMDIS (Automated Mass Spectral Deconvolution and Identification System, NIST, USA) were used. The following mass-spectrometer libraries were applied: NIST2010, the library of the Resource Center of the Science Park “Center for Molecular and Cell Technologies” (St. Petersburg University), the Golm Metabolome Database (GMD) and MoNA (Massbank of North America). Retention index (RI) was determined by calibration with standard alkanes.

Leaf metabolome analysis was performed in three biological and two technical replicates.

### Statistical analysis

All data on plant growth, except that on the number of internodes, and pigment accumulation at stages I–V was processed using two-way analysis of variance (ANOVA) with normal distribution. The multiple comparison procedure was used to isolate which group(s) differ from the others. The data on the number of internodes between cotyledons and the youngest completely formed leaf were processed with one-way ANOVA on Ranks. Data on dry weight (stage VI), mycorrhiza development, and chlorophyll fluorescence were processed with one-way ANOVA with normal distribution. The SPSS 12.0 package (SPSS Inc Chicago, IL, USA) was used for ANOVA. All data were expressed as mean ± standard error. The differences were considered as significant at the confidence level of *p* ≤ 0.05.

Metabolome data were processed in the environment of the R language 3.4.2 (R Core Team, 2017). For quantitative interpretation, the data were normalized against the internal standard (nC23), calculated per mass. In addition, data were normalized against sample median. The data were standardized and log_2_-transformed. Outlying values were excluded on the basis of Dixon’s test. When a metabolite was not detected but was present in the other replicated samples it was considered a technical error and the missing values were imputed. Missing data imputation was performed by KNN (k-nearest neighbors) with “impute” R package (Hastie et al., 2017). A heatmap was constructed with ComplexHeatmap (Gu, Eils & Schlesner, 2016). PCA (Principal Component Analysis, PCA) was realized with pcaMethods (Stacklies et al., 2007). LLE (Locally Linear Embedding) was performed with RDRToolbox (Bartenhagen, 2014). Random Forest method (RF) was carried out in the randomForest toolkit (Liaw & Wiener, 2002), while (O)PLS-DA was performed in Ropls toolkit. Variable Importance in Projection (VIP) was used as a statistic for the feature selection (Thevenot et al., 2015). Non-parametric multivariate analysis of variance (PERMANOVA) (Anderson, 2001) was used with Vegan (Oksanen et al., 2018). Euclidean distances were applied. A hypergeometric test was used to perform an enrichment analysis (Kachitvichyanukul & Schmeiser, 1985). Lists of metabolites tested for overrepresentation was made by OPLS-DA (VIPs ≥ 1) and Random Forest (Mean Decrease Accuracy). The KEGG database (Kanehisa et al., 2019) was used by the R package KEGGREST that provides a client interface to the KEGG REST server (Tenenbaum, 2018). The lists of metabolic pathways which include identified metabolites were obtained using KEGGREST. *M. truncatula* was used as a reference organism, because it is closely related to *P. sativum*. For the compounds identified up to class (hexose, disaccharide, among others), lists of metabolic pathways for common compounds of these classes were used. Results were visualized as the networks of metabolic pathways, where nodes (pathways) share common edge if they include common metabolites. A graph was built in the Cytoscape software environment (Shannon et al., 2003) using Prefuse Layout. Lengths of edges reflect the number of metabolites shared between pathways. Metabolites with significant (*p* < 0.05) and strong correlation coefficients (|*r*| > 0.8) of their arbitrary content were mapped in the Cytoscape software environment (Shannon et al., 2003), using “organic layout.”

## Results

### Arbuscular mycorrhiza development and its effect on host plant growth and physiological state

**Figure 1:**
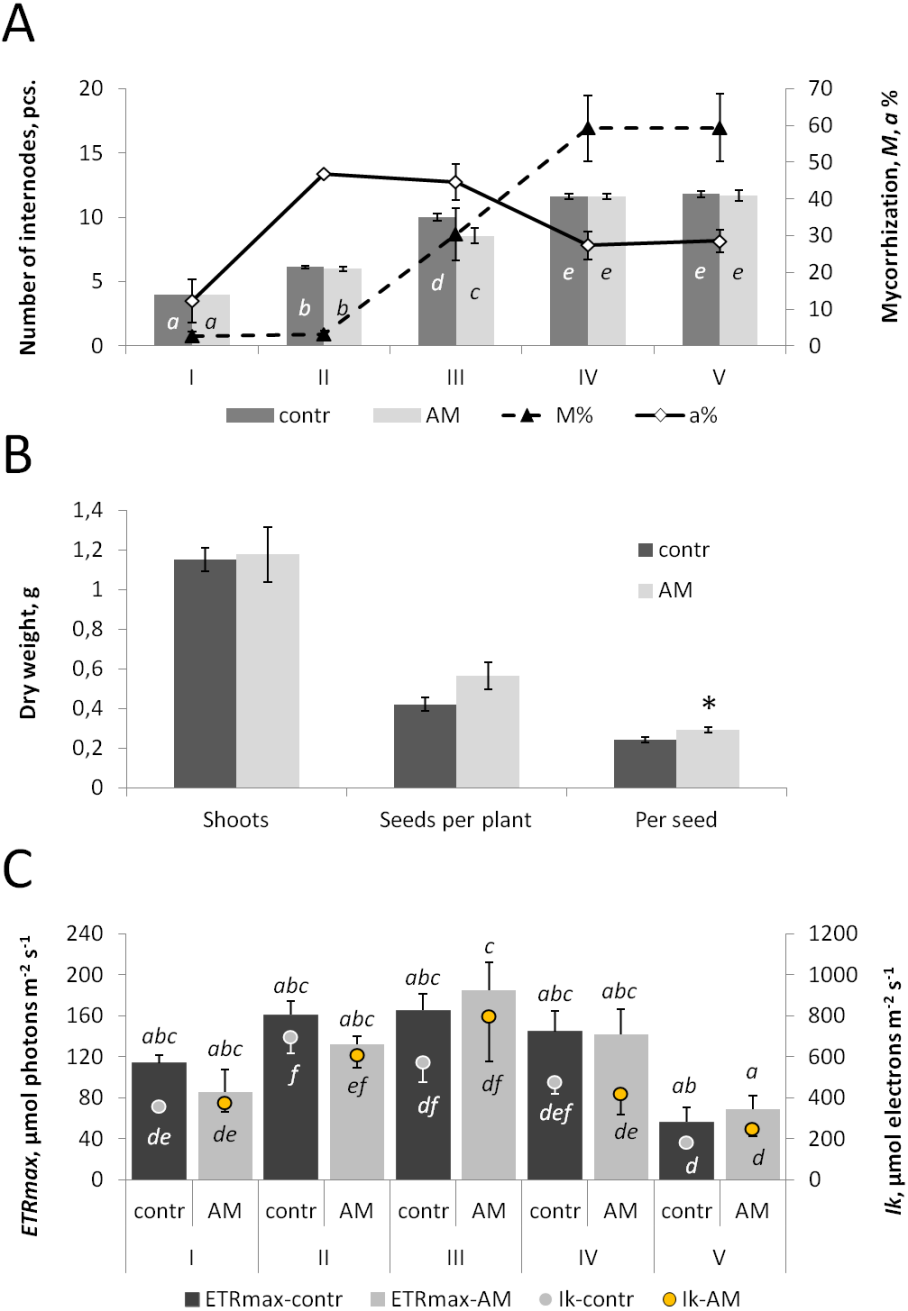
The effect of inoculation with *R. irregularis* on plant growth and leaf photochemical activity of the pea cv. Finale at different stages of plant development. The stages are: I: 7 days post inoculation (DPI) when the second leaf is fully unfolded with one pair of leaflets and a simple tendril; II: 21 DPI at first leaf with two pairs of leaflets and a complex tendril; III: 32 DPI when the floral bud is enclosed; IV: 42 DPI at the first open flower; V: 56 DPI when the pod is filled with green seeds; and VI: 90-110 DPI at the dry harvest stage. Bars represent standard errors. *p* ≤ 0.05. (A) The number of internodes between cotyledons and the youngest completely formed leaf (pcs.) and parameters of arbuscular mycorrhiza development in the inoculated plants: *M*% is the intensity of intraradical mycelium development (no differences were observed between the mean values for stages I and II, as well as between those for stages IV and V), *a*% is the arbuscule abundance in mycorrhizal root fragments (no differences found between the mean values for the stages II and III, as well as between those for stages IV and V). (B) Average dry weight (g) 1) of shoots 2) of seeds per plant 3) per seed (at stage VI). (C) Photochemical activity of the youngest completely developed leaf (one leaflet). *Ik* is the light saturation and minimum saturating irradiance, *ETRmax* is the maximum electron transport rate. (A, C) The values, which are not significantly different from each other, are marked with the same letter. (B) * indicates a significant difference between control and inoculated plants. *contr* signifies control plants, *AM* signifies plants inoculated with *R. irregularis*.

In the plants inoculated with *R. irregularis*, the intensity of intraradical mycelium development (*M*%; Fig. 1A) increased up to stage IV of plant development (see ‘Experimental design and collection of plant material’ for description of the developmental stages) and then plateaued. The most intensive growth of *M*% was observed in between stages II and IV. Arbuscule abundance in mycorrhizal root fragments (*a*%; Fig. 1A) began to rapidly increase at stage I, reached the maximum at stages II–III and then decreased, reflecting a reduction of the functional activity of mycorrhiza. Mycorrhiza was not found in the roots of non-inoculated plants, which indicated that the growth substrate was well sterilized and there was no cross-contamination of the plants during vegetation.

During stages I–V, inoculation with *R. irregularis* had no influence on either the shoot weight or the total fresh weight of cv. Finale pea plants (Table 1). No significant interaction between the factors ‘Stage’ and ‘Inoculation’ was observed (Table 1). At stage III, found to be the most active phase of mycorrhiza development, the number of internodes between cotyledons and the youngest completely formed leaf was lower in the inoculated plants (Fig. 1A). At the same time, correlations between the mycorrhization parameters and the number of internodes in individual plants at this developmental stage (*p* = 0.363 and 0.988, correspondingly, for *M*% and *a*%) were not significant. This growth parameter increased up to stage IV and then stabilized (Fig. 1A) in accordance with the previous characterizations of cv. Finale as exhibiting determinate stem growth (Engvild, 1987).

**Table 1:** Probability (*p*) values that reflect influence of factors ‘Stage’ and ‘Inoculation’ on plant growth and pigment accumulation in the youngest completely developed leaf of the pea cv. Finale. *p* values were calculated using two-way ANOVA. For pairs with *p* ≤ 0.05, the influence of the factor was considered significant (bold and underline). Non-bold underlined p values indicate a marginally non-significant trend of Inoculation factor influencing leaf chlorophylls content (*p* ≤ 0.1).

Parameter	Factor		
	Stage	Inoculation	Stage × inoculation
Shoot fresh weight	$\underline{\lt 0.001}$	0.106	0.721
Total fresh weight	$\underline{\lt 0.001}$	0.443	0.976
Chlorophyll *a*	0.182	$\underline{0.080}$	0.909
Chlorophyll *b*	0.247	$\underline{0.069}$	0.912
Carotenoids	$\underline{\mathbf{0.026}}$	0.108	0.793

The vegetation of the control plants lasted, on average, 90 days, while in the inoculated plants it continued for 2 to 3 weeks longer. Despite the fact that the shoot dry weight was the same with and without AM, inoculation led to an increase of the dry weight per seed (Fig. 1B). Additionally, a marginally non-significant trend towards the increase of the seed dry weight per plant (*p* = 0.054, *n* = 29) was observed.

**Figure 2:**
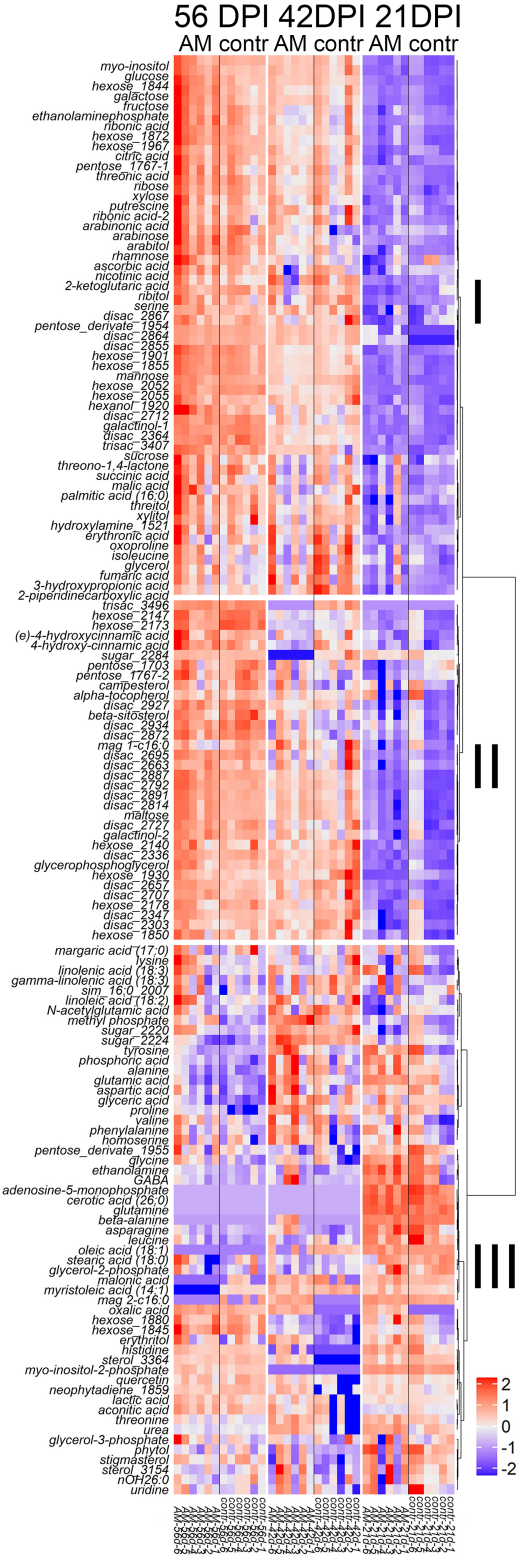
Heatmap of the arbitrary content of identified (at least up to class) metabolites in the youngest completely developed leaf of the pea cv. Finale at different developmental stages. The heatmap is combined with a hierarchical clustering dendrogram (distances represent the correlation of the metabolite concentrations, Ward method). Data are normalized per sample median, log_2_-transformed and standardized. The number after ‘_’ is the retention index (RI), *mag*, monoacylglycerol, *disac*, *trisac*, di and trisacharides, or their derivates. *contr*, control plants, *AM*, plants inoculated with *R. irregularis*. Stage descriptions given in Fig. 1.

Further on, a measurement of the mycorrhization effect on the photochemical activity of pea leaves, chlorophyll *a* fluorescence in a leaflet of the youngest completely formed leaf was taken (Fig. S1). Both the maximum photochemical efficiency of PSII in the darkness-adapted state, *F*_*v*_∕*F*_*m*_, and the effective quantum yield of photochemical energy conversion in PSII, *Y(II)*, were generally constant during vegetation (stages I–V). An exception to this was *Y(II)* at stage I for inoculated plants, which was less than the corresponding value for *F*_*v*_∕*F*_*m*_ (Fig. S2A). The coefficient of photochemical quenching of chlorophyll fluorescence, *qP*, was also constant during plant development, while the coefficient of non-photochemical quenching of chlorophyll fluorescence, *qN*, changed with time relative to *qP* (Fig. 2SB). The maximum electron transport rate at light saturation, *ETRmax*, was constant in the control plants, whereas the inoculated plants had a difference of *ETRmax* values corresponding to stages III and V; namely, at stage III it was higher (Fig. 2C). The minimum saturating irradiance, *Ik*, changed during vegetation (Fig. 2C). In the intermediate developmental stages, *Ik* was higher than in the later stage V for both treatments. In the control, the distinction was more pronounced: differences were also observed between the middle stages and stage I. At the same time, there were no differences between the control and inoculated plants in any of the six characterized parameters of chlorophyll *a* photochemical activity at each of the developmental stages (Fig. 1C; Fig. S2). Thus, despite some differences between control and mycorrhizal plants in the dynamics of changes of these parameters, it can be concluded that mycorrhization, in general, did not affect the photochemical activity of chlorophyll *a* in the leaves of the pea genotype Finale.

Although a pair wise comparison of chlorophyll *a* and *b* accumulation did not reveal significant differences between the control and inoculated plants (Fig. S3), there was a marginally non-significant trend of ‘Inoculation’ factor positively influencing both parameters (Table 1). At the same time, chlorophyll *a* and *b* accumulation values were not dependent on the stage of plant development, unlike the accumulation of carotenoids (Table 1; Fig. S3). A multiple comparison procedure revealed that this parameter differed only for the inoculated plants between stages I and V (Fig. S3). However, inoculation had no effect on the carotenoid content (Table 1; Fig. S3).

Since it was shown that in the model plant *M. truncatula* mycorrhization could lead to an increase in the intensity of photosynthesis by increasing the leaf surface area (Adolfsson et al., 2015), the area of the leaflet used for chlorophyll fluorescence analysis was measured. However, leaflet areas (measured at stages II and V) did not differ significantly in control and inoculated plants and were, respectively, 4.32 ± 0.93 and 5.24 ± 0.27 cm^2^ (for stage II, *p* = 0.119) and 2.84 ± 0.05 and 2.52 ± 0.54 cm^2^ (for stage V, *p* = 0.641).

### Leaf metabolome

#### General characteristics of the metabolite profile

The metabolite profiles of pea leaves included about three hundred metabolites, for half of which the metabolite class was identified (Fig. 2 and Tables S1, S2). Twenty one amino acids (18 proteinogenic), more than ten carboxylic acids, mainly energy metabolism intermediates, 13 fatty acids and their derivatives, as well as nitrogenous bases, sugar alcohols, sterols, various secondary metabolites and others were detected. Sugars (about 70), including pentoses, hexoses and oligosaccharides, were the most widely represented metabolite group in the obtained profiles.

Alterations in the metabolite-relative abundances were visualized as a heat map combined with a hierarchical cluster dendrogram (Fig. 2). The metabolites formed three large clusters. Metabolites of the first (I) and second (II) clusters (Fig. 2) are characterized by a higher content at the later stages of development and, vice versa, metabolites of the third (III) smaller cluster received better representation at earlier stages. The first and second clusters contained a large number of sugars. The first, larger, cluster primarily included monosaccharides. In addition, a number of key organic acids was included in this cluster. The second cluster included, for the most part, metabolites with a high content on the last stage of development, and among them disaccharides were the most abundant. In the third cluster (Fig. 2), sugars were underrepresented, but the group included the majority of fatty and amino acids. Within these clusters, several smaller groups of metabolites could be distinguished.

It was shown that pattern of correlations for metabolite content was very similar in both the inoculated and control plants. A linear relationship and strong correlation between Pearson coefficients in control and inoculated plants was found (*r* = 0.75). On the other hand, the number of strong correlations increased under mycorrhization. The median number of strong correlations (|*r*| > 0.8) of each metabolite was 18.5 for control and 22 for inoculated plants and was statistically (Wilcoxon test) different (*p* = 0.023). This reflected a slight, but significant (*p* < 10^−15^) increase in the level of mean absolute correlation between inoculated (0.434) and control plants (0.418).

**Figure 3:**
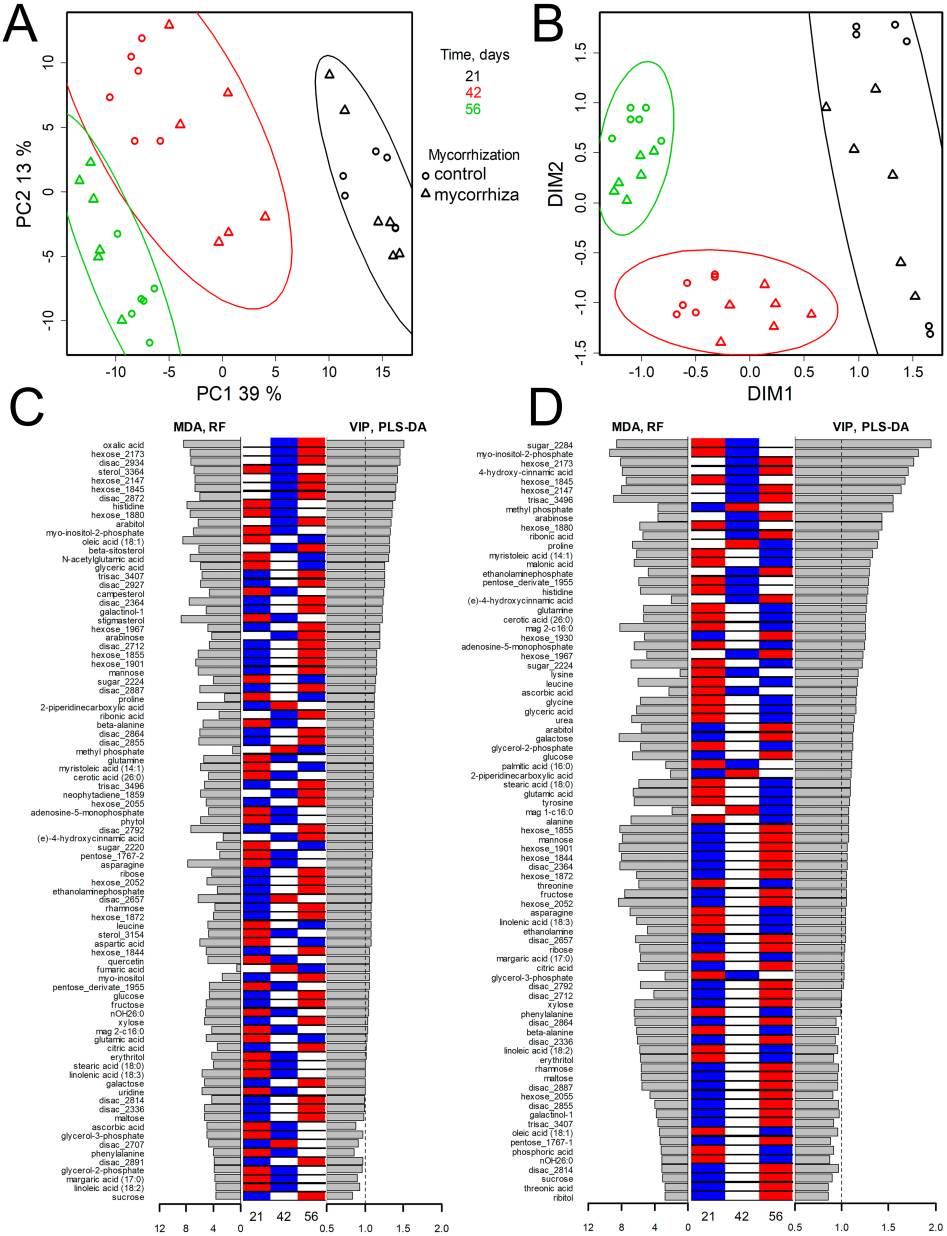
Age dependent metabolome shifts in pea leaves of cv. Finale at different stages of plant development. (A, B) Visualization of metabolic profiles in the low dimensional spaces. (A) PCA score plot, % is the variance associated with a PC. (B) Result of the dimension reduction by Local Linear Embedding (k = 18). Ellipses—95% CI. (C, D) Supervised selection of features related to aging: barplots of Mean Decrease Accuracy from Random Forest and VIPs from PLS-DA combined with heatmap of means for every age for control plants (C), and plants inoculated with *R. irregularis* (D). Stage descriptions are given in Fig. 1.

##### Analysis of alterations in leaf metabolome during plant development.

Differences in pea leaf metabolomes were analyzed in lower dimension planes as obtained with two unsupervised dimension reduction methods (Lee & Verleysen, 2010): the principal component analysis (PCA)—most common in metabolic studies—and LLE (Locally Linear Embedding). The latter gives advantages in the analysis of data with nonlinear regularities. In Fig. 3A, metabolite profiles are visualized in the score space of the first two principal components (PC) obtained from the PCA of the control and inoculated plants. It can be seen that metabolomes were grouped along PC1 in accordance with the plant age (ANOVA *p* = 2.2 × 10^−16^, a Tukey test gives *p* values <10^−13^ for all pairs). In addition, as can be seen in Fig. 3A, an effect of mycorrhization was much weaker, but clearly distinguished at both stages IV (*t*-test for PC1 gives *p* = 0.0016) and V (*p* = 0.0003). A similar result was obtained by LLE application (Fig. 3B) (MANOVA for first two dimensions *p* < 10^−13^), but in this case the effect of mycorrhiza was even more pronounced. For stage IV differences in the first dimension (*p* = 0.003) and for stage V in the second dimension (*p* = 0.0004) were observed. Also PERMANOVA confirmed effects of development stage (*p* = 10^−15^) and mycorrhization ( *p* = 0.04) on the metabolome. It should be noted, that the leaf metabolic profiles of the inoculated plants at stages IV and V were shifted towards those of the control plants at earlier developmental stages (Fig. 3). This implies that mycorrhization slightly retarded plant development.

Further detailed analysis of age dependence of metabolite profiles diversity was done with classification by supervised methods such as PLS-DA and Random Forest. The PLS-DA (Fig. S4 ) model made for the control plants contained two PCs, explaining 41 and 21% of the variance, respectively, at *R*^2^*Y* = 0.99, *Q*^2^*Y* = 0.96 (Fig. S4A). For both control and inoculated plants, plants that were 21 days old were distinguished from the others by an association with PC1, while those between 42 and 56 days old were distinguished by an association with PC2 (Fig. S4A). This indicates a larger metabolic shift between 21 and 42 days compared with that of between 42 and 56 DPI.

Histograms of VIP and MDA (Figs. 3C and 3D) values, which reflect the relation of metabolite content to the age of the plant, are shown in Fig. S4B, S4C. The age of plants strongly affected the content of sugars, including a wide spectrum of disaccharides, such as sucrose, as well as pentoses and hexoses, such as fructose and glucose. According to the heatmap (Figs. 2, 3C and 3D), sugars were accumulated mainly at the age of 56 days. On the other hand, a number of amino acids (e.g., proline, glutamate, glutamine, histidine and asparagine) showed high values of VIP and MDA, but their maximum abundance was observed at the beginning of plant development.

##### The effect of mycorrhization on the leaf metabolome.

A similar analysis was performed on the leaf metabolomes of the inoculated plants (Fig. S4 ). To evaluate the similarities and differences in the metabolomic shifts during aging of the control and inoculated plants, loadings of the first and second components of the PLS-DA models were compared. Scatter plots in the spaces of the loadings of the PCs of the PLS-DA models were created for analysis. In the case of the PC1 (Figs. S4C), which in both models were associated with differences in the profiles of plant metabolites at the 21st day of development from the others, the dependence of their loadings was close to linear and the values were very similar. The correlation coefficient was *r* = 0.93 and highly significant (*p* < 10^−15^).

According to the loadings plot for the PC2 (Fig. S4D), which was related to differences between 42 and 56 DPI, a correlation was also estimated. It was not so strong (*r* = 0.49) but significant (*p* < 10^−15^). Thus differences between inoculated and control plants were much greater at the later stages. In the case of control plants, a number of metabolites (in the higher left sector of Fig. S4D) decreased or did not show significant change at the last stage, but they stayed at the same level or increased in the leaves of inoculated plants. Several amino acids, intermediates of nitrogen metabolism, and unsaturated fatty acids increased their content under mycorrhization. In contrast, several sugars and sterols (in the right lower section of Fig. S4D) accumulated in the control plants and either decreased or did not change their level, or accumulated at a lesser rate under inoculation. This is consistent with the “shift” of the 56 day old plants in the direction of 21 day old plants along PC2 at the PLS-DA score plot (Figs. S4A, S4B). Thus, mycorrhiza modifies specific segments of metabolic network at later stages of plant development, making inoculated plants at stages II and V metabolically closer to each other, at least partially. Moreover, mycorrhiza slightly retards age-related metabolome alterations as can be seen above at the PCA and LLE plots (Figs. 3A 3B). As presented at Figs. S5C, S5D, the samples of inoculated plants at later stages are shifted toward samples of the preceding time points. In particular, there is a quite recognizable shift from 42 DPI plants to 56 DPI plants along PC1 on the PLS-DA score plot (Fig. S5C).

In order to find out which pathways were the most affected by aging an enrichment analysis was performed. A list of significant features was identified with PLS-DA and Random Forest (Fig. 3C). As was expected according to the literature, plant age yielded the greatest effects on sugar metabolism, including galactose metabolism (*p* = 0.037), starch and sucrose metabolism (*p* = 0.031), amino sugar and nucleotide sugar metabolism (*p* = 0.035).

Moreover, the alterations of sterol metabolism (*p* = 0.06), the components of lipids, were also associated with plant age. Thus, plants at different stages of development demonstrate systemic differences based on rearrangements in the metabolic pathways primarily associated with carbohydrate metabolism, possibly including photosynthesis, carbon transport and storage.

**Figure 4:**
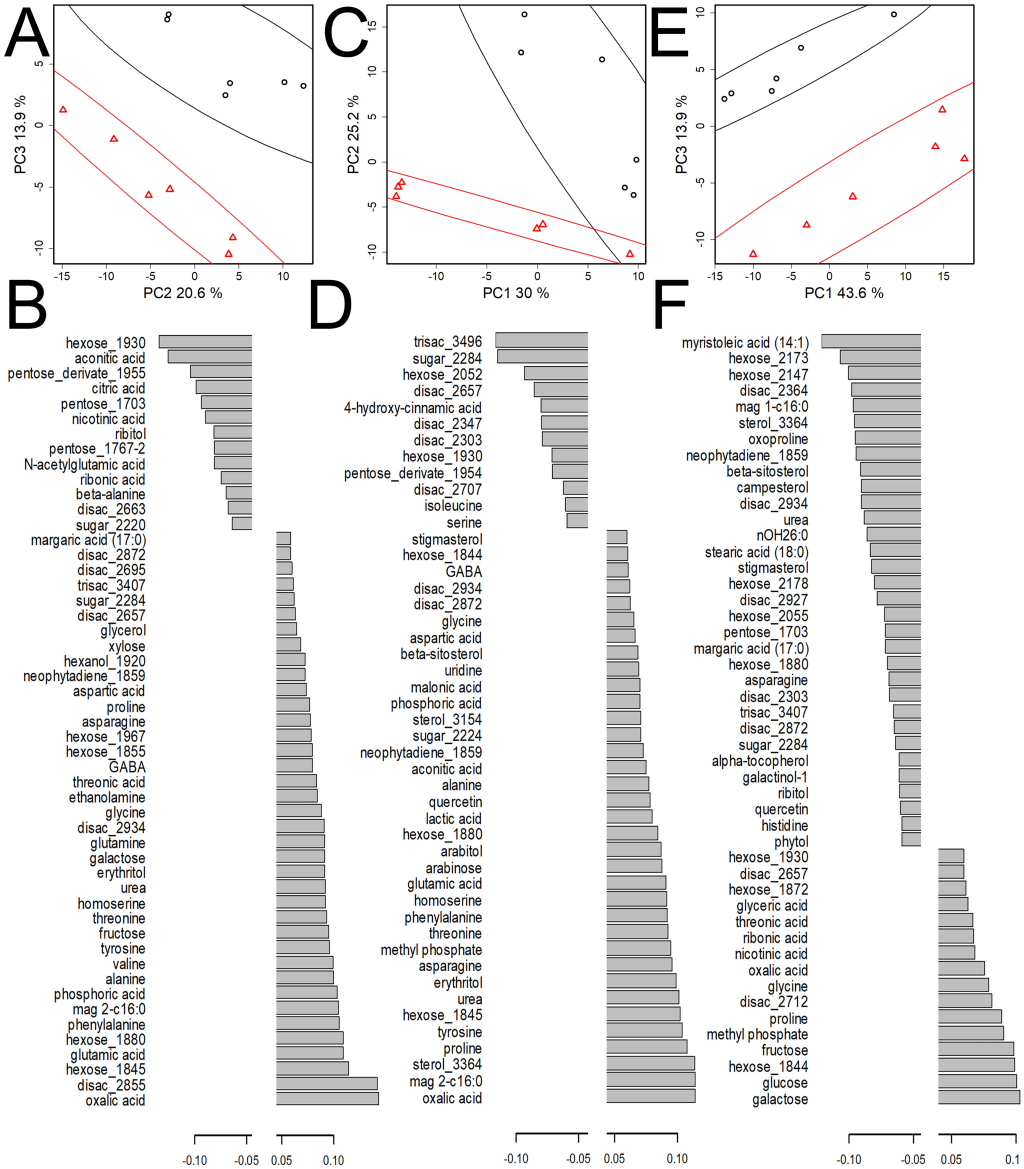
Comparative analysis of metabolite profiles of mycorrhizal and control pea plants of cv. Finale at different stages of plant development. (A, B) 21 DPI, stage II; (C, D) 42 DPI, stage IV; (E, F) 56 DPI, stage V. (A, C, E) PCA score plot, % is the variance associated with the PC. Ellipses—95% CI, circles represent control, the triangles represent the mycorrhizal plants. (B, D, F) Diagrams of loadings of the OPLS-DA predictive component (VIP > 1). Negative values correspond to a higher content in control plants and vice versa. Stage descriptions given in Fig. 1.

#### The effect of inoculation with *R. irregularis* on the leaf metabolite profile at different developmental stages

##### Differences at stage II (21 DPI).

According to unsupervised dimension reduction by PCA (Fig. 4A) and LLE (Fig. S6A) the samples clustered in relation to mycorrhization status. With PCA, a clear difference was found in the space of PC2 and PC3 (MANOVA *p* = 5.24 × 10^−7^). The PERMANOVA for nonreduced data gave *p* = 0.099. In order to determine the details of the differences in the metabolic profiles of the control and inoculated plants, classification by OPLS-DA was carried out. Sixteen percent of variation in metabolite content was associated with the predictive component, with *R*^2^*Y* = 0.99, *Q*^2^*Y* = 0.86. Thus, during this period, the role of mycorrhization in the formation of the metabolite profile of leaf was significant, but relatively minor. Figure 4B shows the plot of the predictive component loadings with VIPs >1. Negative loadings correspond to a higher content in control plants and vice versa. As can be seen, a greater number of metabolites demonstrates a higher content in inoculated plants. Values of loadings (Fig. 4B) show that the inoculated plants contained more amino acids such as glutamate, glutamine, glycine, homoserine, phenylalanine, proline, threonine, valine. Also in the leaves of the inoculated plants, the content of other nitrogen-containing compounds is higher, including such important metabolites as urea and GABA. At the same time, the leaves of the control plants contain more β-alanine, serine and N-acetylglutamate. On the other hand, in the control plants, a higher content of TCA intermediates such as citrate and aconitate was observed. Accumulation of carbonic acids was associated with inhibition of synthesis of other amino acids as well as with a more active catabolism and urea cycle. Various sugars accumulated in both control and inoculated plants. Fatty acids did not show a clear dependence on mycorrhiza in this period. It should be noted that higher content of phosphate in the leaves was observed in the inoculated plants, which may be the result of an advantage in mineral nutrition.

Enrichment analysis (list of metabolites with VIP >1) for 21 day old plants showed that seven pathways were significantly affected by the mycorrhization. Four of them are related to amino acid metabolism: Alanine, aspartate and glutamate metabolism (*p* = 0.083), Aminoacyl-tRNA biosynthesis (*p* = 0.036), Biosynthesis of amino acids (*p* = 0.014), Cyanoamino acid metabolism (*p* = 0.074). Thus nitrogen metabolism and various amino acids were primarily affected. Also 2-Oxocarboxylic acid metabolism (*p* = 0.090), Pantothenate and CoA biosynthesis (*p* = 0.082), and Porphyrin and chlorophyll metabolism (*p* = 0.082) were affected by mycorrhiza.

*Differences at stage IV (42 DPI).* As in the case of 21 day old plants, unsupervised dimension reduction (Figs. 4C, S6B) showed that the samples grouped according to the mycorrhization status. But in this case PCA, in contrast to 21 DPI, stood out in the space of PC1 and PC2 (MANOVA *p* = 0.0005). PERMANOVA for nonreduced data gave *p* = 0.004. Thus, the effect of mycorrhiza was more pronounced than at stage II. OPLS-DA showed that 25% of the variance of the metabolites content was associated with the predictive component, and *R*^2^*Y* = 0.94, *Q*^2^*Y* = 0.87. Thus, for this period, the role of mycorrhization in leaf metabolome formation increased as compared to that of 21 day old plants. Considering the loadings of the predictive component (Fig. 4D), it can be seen that positive values corresponding to a higher content in inoculated plants were characteristic for a larger number of metabolites. The differences in the metabolic profiles of inoculated and control plants had a number of common features at 21 and 42 DPI. After six weeks of mycorrhization, leaves showed higher levels of a number of amino acids and intermediates of nitrogen and phosphorus metabolism. Organic acids such as aconitate, lactate, malonate, among others, also increased. The content of some sterols also became higher at 42 DPI in the inoculated plants. During this period the list of metabolites, whose content was higher in the leaves of the control plants, mainly contained sugars. Exceptions were the amino acids serine (as on day 21), and isoleucine. Differences were found in the content of secondary metabolites, for example, 4-hydroxycinnamic acid.

The effects of aging and mycorrhization were compared at 42 DPI. A scatter plot was created in the spaces of the loadings of the predictive components from the two OPLS-DA models: one for comparing the 21 and 42 DPI plants, and the other for the control and inoculated plants at 42 DPI (Fig. S5A). Values of loadings of predictive components from these two models are strongly correlated. The correlation coefficient was *r* =  − 0.61 and highly significant (*p* < 10^−15^). Thus, in general, mycorrhization has an opposite effect on metabolite content to that of aging. In addition, the ‘rejuvenating’ effect of mycorrhization clearly appeared on the score plot (produced by PLS-DA) of the 42 DPI plants of both the control and the treatment groups and the 21 DPI control plants (Fig. S5C). As can be seen, the inoculated plants at 42 DPI are closer to the 21 DPI control plants than the 42 DPI control plants.

**Figure 5:**
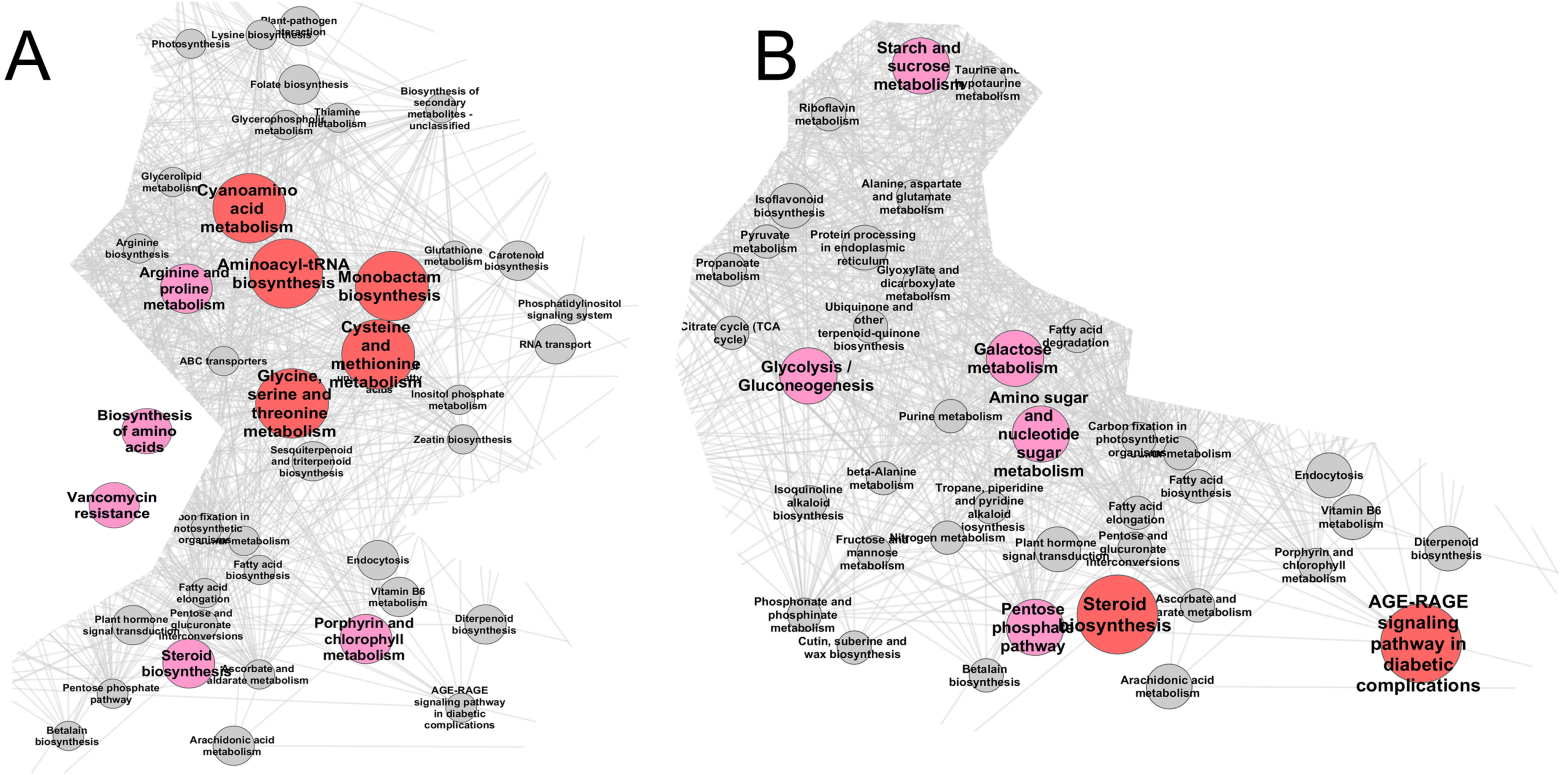
The effect of mycorrhiza development revealed by enrichment analysis (hypergeomertric test) on the metabolic pathways in the pea leaves of cv. Finale. Fragment of pathway network based on the KEGG database using *Medicago truncatula* as a reference species. Nodes (pathways) share common edge if they share metabolites. The graph was built in the Cytoscape using Prefuse Layout, where lengths of edges reflect the number of metabolites shared between pathways. The larger red nodes correspond to *p* < 0.05, smaller pink nodes to *p* < 0.1, grey nodes to pathways sharing metabolites with significantly affected ones. (A) 42 DPI (stage IV); (B) 56 DPI (stage V). Full versions of the graphs are shown in the Fig. S6.

Enrichment analysis (Figs. 5, S7) showed that the range of pathways, which were affected by mycorrhization at the 42nd day, expanded compared to the 21st day and included (Fig. 5A, Fig. S4A): Aminoacyl-tRNA biosynthesis (*p* = 0.20), Arginine and proline metabolism (*p* = 0.091), Biosynthesis of amino acids (*p* = 0.060), Cyanoamino acid metabolism (*p* = 0.009), Cysteine and methionine metabolism (*p* = 0.027), Glycine, serine and threonine metabolism (*p* = 0.043), monobactam biosynthesis (*p* = 0.027), Porphyrin and chlorophyll metabolism (*p* = 0.066), Steroid biosynthesis (*p* = 0.091), Vancomycin resistance (*p* = 0.066). Figs. 5A and S7A show that mycorrhization primarily affects segments of metabolic network related to amino acid and protein biosynthesis, and lipophilic compound metabolism.

##### Differences at stage V (56 DPI).

Just as with the 21 DPI and 42 DPI plants, dimension reduction showed (Figs. 4E, S6C) that samples grouped according to mycorrhization status. In this case a clear difference was observed in the space of PC1 and PC3 (MANOVA *p* = 1.2 × 10^−7^). The PERMANOVA for nonreduced data gave *p* = 0.008. The OPLS-DA showed that 23% of the variance of the content of metabolites was associated with the predictive component, with *R*^2^*Y* = 0.99, *Q*^2^*Y* = 0.86. Thus, during this period, mycorrhization exerted the same influence on the formation leaf profile metabolites as that at 42 DPI. Analysis of the predictive component loadings (Fig. 4F) showed that negative values corresponded to a higher metabolite content in control plants where a larger number of metabolites was observed. Lipophilic compounds, sterols, terpenes, and fatty acids were among the metabolites with higher content in the leaves of control plants. Several intermediates of TCA demonstrated the same trend. At the same time there were no fatty acids, sterol or acylglycerol among metabolites which demonstrated higher content in the inoculated plants. Interestingly, major monosaccharides and few amino acids also demonstrated positive factor loadings. Comparative analysis of the aging and mycorrhiza effects (in the same way as for 42 DPI) revealed (Figs. S5B, S5D) that these two factors have the opposite effect (*r* =  − 0.37, *p* < 10^−10^).

Enrichment analysis (Figs. 5B, S4B) showed that the range of pathways affected by mycorrhization during this period was very different from that at 42 and 21 DPI. At the same time, the mycorrhiza-affected pathways were similar to those affected by aging. Changes in the metabolism of sugars, including glycolysis and lipophilic compounds were more pronounced. The following pathways were affected: AGE-RAGE signaling pathway in diabetic complications (*p* = 0.048), Amino sugar and nucleotide sugar metabolism (*p* = 0.069), Galactose metabolism (*p* = 0.067), Glycolysis/Gluconeogenesis (*p* = 0.067), Pentose phosphate pathway (*p* = 0.079), Starch and sucrose metabolism (*p* = 0.079), Steroid biosynthesis (*p* = 0.031).

## Discussion

The main goal of the present study was to reveal whether and how mycorrhization affects growth and metabolism of the aerial part of pea plants at the key plant developmental stages. For this purpose gas chromatography-mass spectrometry (GC-MS) was used (Van Look, Simchen & Heberle, 1995; Lisec et al., 2006; Puzanskiy et al., 2018). The youngest completely developed leaf was studied, with the assumption that it had the most active metabolism. The study also included analyses of its photochemical activity and pigment accumulation, which showed that AM development did not affect the physiological state of the plants. The major achievement of this study is not only that remarkable alterations of the leaf metabolite profile with plant age were demonstrated, but it was also found that mycorrhization can influence age-related changes in the leaf, prolonging the active phase of its metabolism. This is also concurs with the observed retardation of plant growth and delayed senescence in mycorrhizal plants.

### The effect of mycorrhization on plant growth and physiological state

Under the conditions of this experiment, no positive effect of mycorrhiza on the growth parameters of pea during the vegetative and reproductive stages (I-V) was revealed. The data obtained here are consistent with numerous observations made by other authors who noted a low growth response of *P. sativum* to AMF mono-inoculation (Rivera-Becerril et al., 2002; Xavier & Germida, 2003; Borisov et al., 2004b; Desalegn et al., 2016; Zhukov et al., 2017). It was also previously shown that the effect of mycorrhiza was often not manifested under artificial lighting conditions (Konvalinková & Jansa, 2016), so it is likely that the illumination in the growth chambers used in this study was not sufficient for the growth response to occur. Additionally, in the present study, plants were grown under conditions of severe phosphate deficiency; as was shown in previous works under such conditions the plant strongly stimulates root colonization by AMF (Smith & Read, 2008; Bonneau et al., 2013). Taking into consideration the specific conditions, one can presume that excessive colonization by the fungus was not beneficial for the plant at certain developmental stages, since the carbon cost of maintaining the microsymbiont may outweigh the positive effects of mycorrhization.

In the present study, the dynamics of AM development were similar to those for other annual plants. This growth curve rises until plant flowering, and then the fungi stop growing and require carbon only for maintenance. However, the relative costs of AM symbiosis are larger in early plant development when AM fungal colonization is indispensable for plants because the root system is small and hyphae are more efficient in reaching P, which is poorly-mobile in soil (for review, see Kaschuk et al., 2009). This might have led to the observed slight holdback in the formation of stem nodes by the middle of the life cycle in mycorrhizal plants.

Despite the lack of a significant increase in the shoot and seed dry weight per plant under the influence of AMF, the inoculated plants had larger seeds at the dry harvest stage (VI). The inoculated pea plants could probably accumulate additional seed biomass due to an extension of their vegetation period. These observations showed that the inoculated pea plants did have a longer vegetation period, compared with the control plants. Previously, the effect of extending the vegetation period was noted for pea inoculated with both AMF and rhizobia, but no measurements that would confirm retardation in the life cycle of pea plants had been carried out (Naumkina, 2007). In the present study, the pea plants inoculated with AMF prolonged the active phase of the vegetation period, as evidenced by the results of the metabolome analysis of the youngest fully developed leaf (see ‘Influence of the stage of plant development and mycorrhization on the metabolite profile of pea leaves’).

One possibility is that control plants accelerated the completion of their life cycle, since their mineral nutrition probably was worse than that of mycorrhizal plants. It is known that poor nutrition can cause acceleration of flowering and the completion of the life cycle (Takeno, 2016), as well as premature flowering cessation, as shown for pea (Jeuffroy & Sebillote, 1997). Changes in hormonal balance, potentially occurring in inoculated plants, may also contribute to the observed differences. For example, gibberellic acid accelerates flowering (Fleet & Sun, 2005), while abscisic acid detains this process (McCourt & Creelman, 2008).

A similar delay in the aging of inoculated plants has been previously described for *Capsicum annuum*, but there the effect only manifested under salinity stress (Beltrano et al., 2013). In a contrary example, for *Medicago sativa* it was shown that mycorrhization can shorten the vegetation period and at the same time increase the growth parameters of the plant as well as increase photosynthetic acclimation. However, such results were demonstrated only against the background of increased CO_2_ content (Goicoechea et al., 2014). Interestingly, mono-inoculation of pea with rhizobia extended the vegetation period, while simultaneous use of rhizobia and foliar micronutrient fertilizer did not. At the same time, increase of seed yield compared to control was equal in both treatments (Zając, Klimek-Kopyra & Oleksy, 2013). Thus, the change in the vegetation period of the mycorrhizal plants may be associated with both environmental conditions and the individual characteristics of a particular plant genotype.

It is assumed that the enhanced outflow of carbon from the aerial part of a plant into its root system should stimulate the photosynthesis process (Kaschuk et al., 2009). Nevertheless, in the present study, no changes were found to be associated with AMF inoculation, either in the photochemical activity or in the surface area of the leaves. Only a marginally non-significant trend was observed for chlorophyll *a*, *b* accumulation. At the same time, changes in some parameters of photochemical activity at certain stages of development, both in the control and inoculated plants, were revealed indicating normal development and functioning of PSII. Given the fact that enhancement of photosynthesis due to mycorrhization as described in the literature usually occurs under conditions of abiotic stress (Wu & Xia, 2006; Beltrano et al., 2013; Rozpądek et al., 2014; Hashem et al., 2015; Liu et al., 2015; Shinde & Thakur, 2015; Yang et al., 2015; Yooyongwech et al., 2016; Mathur, Sharma & Jajoo, 2018), it can be assumed that mycorrhization by itself may not have direct impact on the function of the photosynthetic apparatus. Rather, it might indirectly affect photosynthesis owing to the overall increase in plant fitness and the increase of its aerial part. The question of how the plant manages to compensate for the carbon outflow from the aerial part to mycorrhizal roots remains.

### Influence of the stage of plant development and mycorrhization on the metabolite profile of pea leaves

The GS-MS profiling of pea leaf revealed more than three hundred substances. Simple unsupervised (PCA, LLE) methods showed significant differences in pea leaf metabolome between different stages of plant growth. These differences were much more pronounced than alterations triggered by mycorrhization. Similar phenomenon of lesser importance of AM formation compared to such factors as age, species specificity, fertilization, season of experiment etc. has been described by other researchers (Fester et al., 2011; Schweiger et al., 2014; Hill et al., 2018).

Further analysis (PLS-DA) indicated more drastic leaf metabolome alterations between stages II and IV in comparison with those between stages IV and V. At stage II plants grew intensively, while at stages IV and V (first open flower and pod fill, green seeds stages) they stopped growing and formed reproductive organs. Thus, plant development was possibly accompanied by drastic changes in patterns of donor–acceptor relations between organs and this coincided with alterations in biochemical pathways.

Enrichment analysis and PLS-DA revealed intensive changes in carbohydrate metabolism. Elevation in sugar content was associated mostly with pea plant aging. Alterations in sucrose, fructose, and glucose might suggest changes in the activity of photoassimilate outflow. The pea plants at the flowering period and, especially, at the pod fill stage were characterized by a high content of disaccharides and hexoses in the leaves, which may be the result of an active synthesis of transport forms of sugars for their subsequent transition to the reproductive organs (Troughton & Currie, 1977).

The sugars with content growing during the flowering and pod fill stages were not limited to sucrose. Unfortunately, these sugars were not identified definitively and thus for the moment their role remains unclear. They might be intermediates of starch or other polysaccharides, containing sugar fragments or transport forms of carbon found in plants (Lalonde, Wipf & Frommer, 2004). Moreover, different transport glycosides of active compounds such as hormones might be among them (Park et al., 2017).

Metabolite profiles of young leaves (stage II) were characterized by higher levels of amino acids. This indicates a higher level of nitrogen metabolism associated with growth in this period. This is consistent with the previously published data concerning raised content of free amino acids in young leaves of *P. sativum* (Storey & Beevers, 1978). Leaves at this age were shown to accumulate higher contents of C14-18 fatty acids, especially unsaturated ones, and their amount decreased with aging. All this could be the result of age-dependent changes in the structure of membranes and lipid metabolism associated with cessation of growth. It is known that the intensity of synthesis of different types of lipids can vary during plant development (Troncoso-Ponce et al., 2013). In pea plants in particular, a change in the activity of the synthesis of both different types of glycerolipids and sterols was reported (Hellgren & Sandelius, 2001). The process slowed down with age. This indicates that the metabolism of leaves of different ages from one plant can vary significantly (Dietz & Heilos, 1990; Desbrosses et al., 2005; Puzanskiy et al., 2018).

Turning back to the role of mycorrhiza effects on the metabolome and possible alterations during development, it should be noted that the observed profile changes were uneven. According to the results of OPLS-DA, the mycorrhiza effect on metabolism was less pronounced at the earlier stage II than at stages IV and V. This phenomenon may be associated with AM colonization (*M%*) (at stage II it was very low whereas at stages IV and V it reached its maximum level). The alterations between stages IV and V were also significantly different between inoculated and control plants. In the process of plant development, the number of metabolites that were more abundant in inoculated plants increased in comparison to control plants. It is known that in some cases mycorrhiza contributes to the accumulation of proteins, carbohydrates, primary and secondary metabolites, probably due to a better supply of phosphate and nitrogen (Fester et al., 2011; Pedone-Bonfim et al., 2013; Goicoechea et al., 2014; Hodge & Storer, 2015); it may also promote photosynthetic plant acclimation at the late stages of vegetation as was shown in *Medicago sativa* and other plants (Goicoechea et al., 2014; De Souza et al., 2016). The observed metabolite shift probably indicates an adaptation of the plant to new nutritional conditions and further redistribution of metabolites in the plant-fungi symbiosis.

The leaves of AM plants contained higher levels of amino acids and unsaturated fatty acids, a fact which coincides with more active metabolism. Sterol levels were accordingly lower than in non-inoculated plants. Thus, mycorrhization partially slows down the aging process and makes the leaf profiles of old mycorrhizal plants a little “younger”. This effect was associated with prolongation of the vegetation period and the increase in seed biomass of the inoculated plants. The present data are consistent with recent discoveries in the field of proteomic research. It was found that at the stage of seed maturation a highly symbiotically effective genotype K-8274 (cv. Vendevil) under combined inoculation with AMF and rhizobia had the proteomic signatures of ongoing seed filling as compared to non-inoculated plants, which shows that inoculation prolongs the active phase of seed filling in some genotypes (Mamontova et al., 2019).

## Conclusions

To the best of our knowledge, this is the first study in which the effect of mycorrhization on the pea leaf metabolome has been examined at different plant developmental stages. Although there was no significant effect of mycorrhization on the aerial biomass, or on the accumulation of chlorophyll *a*, *b* and carotenoids in the leaves or their photochemical activity, it did influence the age-related changes in the plant leaf metabolome. This effect was more pronounced at later stages of plant development. The results of the analysis allow us to conclude that mycorrhization prolongs the period of ‘youth’ in plant leaves, and possibly leads to better accumulation of metabolites such as amino acids and unsaturated fatty acids. Thus, it can be assumed that mycorrhiza partially halts senescence due to a boost of some segments of metabolic network at later stages of plant development. This effect promises to be beneficial for agriculture, especially for northern regions where prolongation of the pea life cycle does not lead to preliminary drying of the seeds and plants in general, and for the green pea cultivars which are harvested before seed maturation.

##  Supplemental Information

10.7717/peerj.7495/supp-1 Figure S1 Measurement of the photochemical activity of pea leaf using a portable chlorophyll fluorometer The arrow points to a leaflet in the first pair of the youngest fully formed leaf. The analyzed leaflet is secured in place using the clamp equipped with a quantum and temperature sensor and connected to the fluorometer.

10.7717/peerj.7495/supp-2 Figure S2 Photochemical activity of the youngest completely developed leaf (one leaflet) of the pea cv. Finale at the different stages of plant development The stages are: I: 7 days post inoculation (DPI) when the second leaf is fully unfolded with one pair of leaflets and a simple tendril; II: 21 DPI at first leaf with two pairs of leaflets and a complex tendril; III: 32 DPI when the floral bud is enclosed; IV: 42 DPI at the first open flower; V: 56 DPI when the pod is filled with green seeds; and VI: 90-110 DPI at the dry harvest stage. The values, which are not significantly different from each other (*p* ≤ 0.05) are marked with the same letter. Bars represent standard errors. (A) *F*_*v*_∕*F*_*m*_, the maximum PSII photochemical efficiency in the darkness-adapted state, *Y(II)*, effective quantum yield of photochemical energy conversion in PSII; (B) *qP*, coefficient of photochemical quenching of chlorophyll fluorescence, *qN*, coefficient of non-photochemical quenching of chlorophyll fluorescence. *contr*, control plants, *AM*, plants inoculated with *Rhizophagus irregularis*.

10.7717/peerj.7495/supp-3 Figure S3 Accumulation of pigments in the youngest completely developed leaf of the pea cv. Finale at the different stages of plant development (see descriptions in Fig. S2) The values for each parameter, which are not significantly different from each other (*p* ≤ 0.05) are marked with the same letter. Bars represent standard errors. *contr*, control plants, *AM*, plants inoculated with *R. irregularis*.

10.7717/peerj.7495/supp-4 Figure S4 Comparative analysis of metabolome shifts during plant development in the pea cv. Finale control and mycorrhizal plants PLS-DA score plots with model parameters for control plants (A) and plants inoculated with *R. irregularis* (B). Scatter plots in the spaces of the PLS-DA loadings of: (C) PC1, (D) PC2.

10.7717/peerj.7495/supp-5 Figure S5 Systemic aging delay in the pea cv. Finale plants inoculated with *R. irregularis* (A, B) Scatter plot in the spaces of the loadings of the predictive components from two OPLS-DA models: (A) First, for comparing 21 and 42 DPI plants; second, for control and inoculated (AM) 42 DPI plants, (B) First, for comparing 42 and 56 DPI plants; second, for control and AM 56 DPI plants. PLS-DA score plots with model parameters for the (C) 42 DPI control and AM and 21 DPI control, (D) 56 DPI control and AM and 42 DPI control.

10.7717/peerj.7495/supp-6 Figure S6 Comparative analysis of metabolite profiles of mycorrhizal and control pea cv. Finale plants at different stages of plant development by using LLE (Locally Linear Embedding) (A) 21 DAI (stage II), *k* = 10; (B) 42 DAI (stage IV), *k* = 6; (C) 56 DAI (stage V), *k* = 10. Black –control, red –plants inoculated with *R. irregularis*, ellipses –90% CI, DIM –dimension.

10.7717/peerj.7495/supp-7 Figure S7 The effect of mycorrhiza development revealed by enrichment analysis (hypergeomertric test) on the metabolic pathways in the pea cv. Finale leaves. Full versions of the graphs shown in Fig. 5 Pathway network based on KEGG database using *Medicago truncatula* as a reference species. Nodes (pathways) share common edge if they share metabolites. Graph was built in the Cytoscape environment using Prefuse Layout, where lengths of edges reflect the number of metabolites shared between pathways. The bigger red nodes correspond to *p* < 0.05, smaller pink nodes to *p* < 0.1, grey nodes to pathways sharing metabolites with significantly affected ones. (A) 42 DPI (stage IV); (B) 56 DPI (stage V).

10.7717/peerj.7495/supp-8 Table S1 Metabolite content, a. u. determined as peak areas (sum for isomers) normalized by area of internal standard (tricosane, 20 µg) peak area and normalized per weight Abbreviations for compound names: *mag*, monoacylglycerol; * disac, trisac*, unidentified di- or trisacharide or derivate; *sim*, similar to; *ni*, unidentified compounds; *RI*, retention index; _number is RI for compounds identified just up to class. *AM*, plants inoculated with *R. irregularis*, *DPI*, days post inoculation. Bold type marks values imputed by KNN, see materials and methods

10.7717/peerj.7495/supp-9 Table S2 Metabolite content normalized by sample (row) median Abbreviations for compound names: *mag*, monoacylglycerol; *disac, trisac*, unidentified di- or trisacharide or derivate; *sim,* similar to; *ni*, unidentified compounds; *RI*, retention index; _number is RI for compounds identified just up to class. *AM*, plants inoculated with *R. irregularis*, *DPI*, days post inoculation. Bold type marks values imputed by KNN, see materials and methods.

10.7717/peerj.7495/supp-10 Supplemental Information 1 Raw GC-MS data presented in chromatograms for metabolite content in the leaves of control and mycorrhizal pea plants (21 days post inoculation, stage II)

10.7717/peerj.7495/supp-11 Supplemental Information 2 Raw GC-MS data presented in chromatograms for metabolite content in the leaves of control and mycorrhizal pea plants (42 days post inoculation, stage IV)

10.7717/peerj.7495/supp-12 Supplemental Information 3 Raw GC-MS data presented in chromatograms for metabolite content in the leaves of control and mycorrhizal pea plants (56 days post inoculation, stage V)

## Additional Information and Declarations

### Data Availability

The following information was supplied regarding data availability: The raw data are available in the Supplemental Files.
