# Supplementary figures and images for: Metabolic alterations in pea leaves during arbuscular mycorrhiza development
Source: PeerJ. 2019 Aug 23;7:e7495. doi: 10.7717/peerj.7495 (PMC6709666; doi:10.7717/peerj.7495)

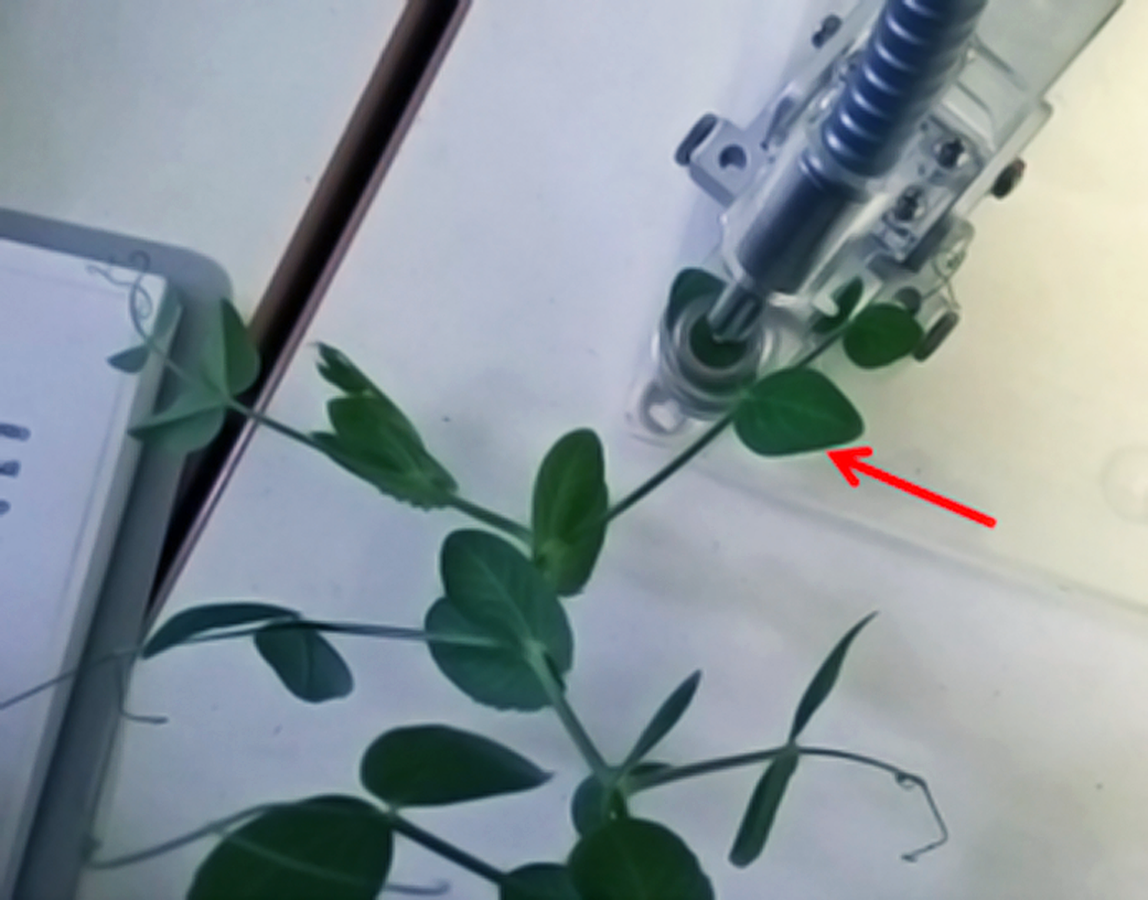

Supplement: Figure S1 — The arrow points to a leaflet in the first pair of the youngest fully formed leaf. The analyzed leaflet is secured in place using the clamp equipped with a quantum and temperature sensor and connected to the fluorometer. [file peerj-07-7495-s001.png]

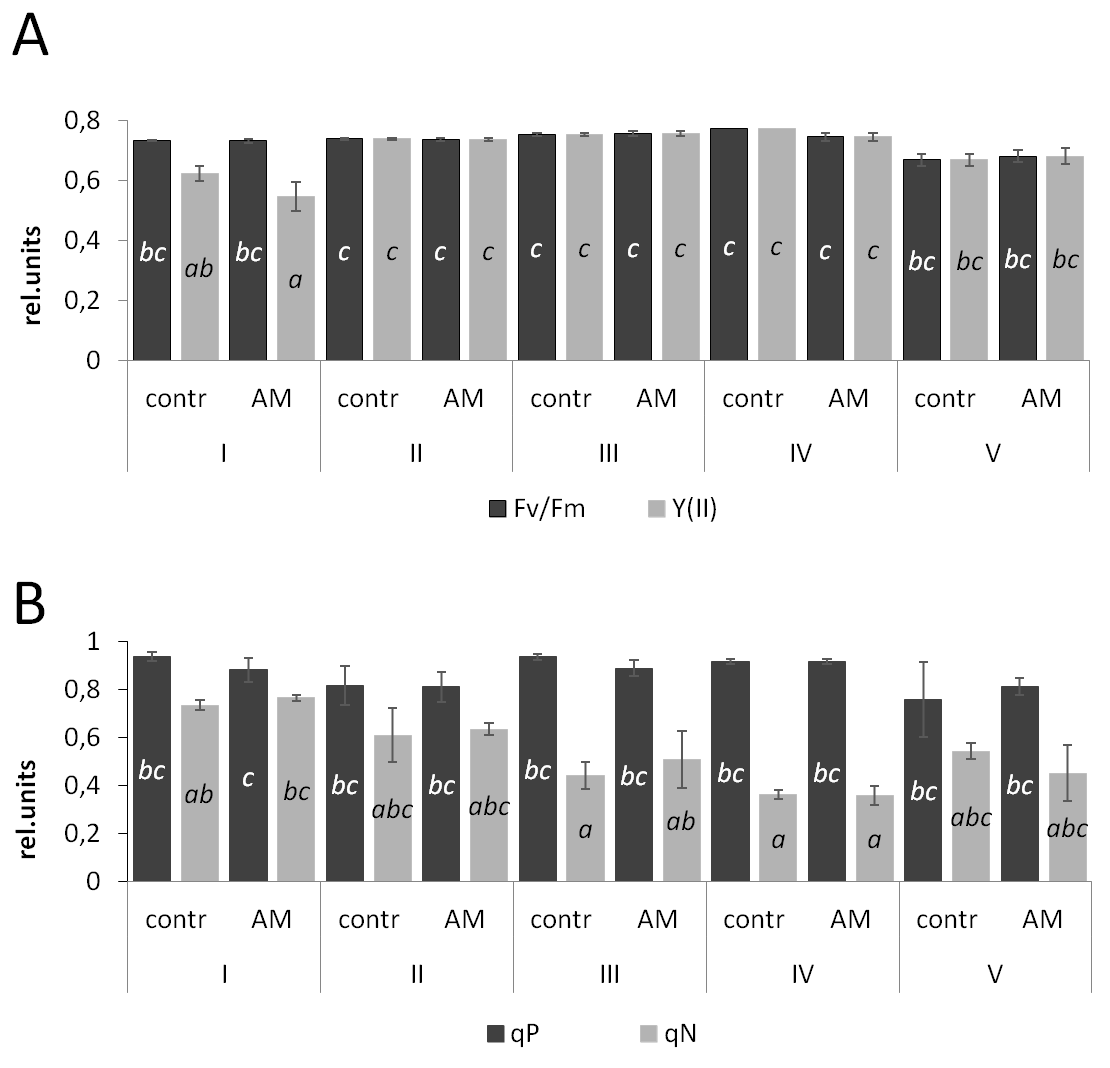

Supplement: Figure S2 — The stages are: I: 7 days post inoculation (DPI) when the second leaf is fully unfolded with one pair of leaflets and a simple tendril; II: 21 DPI at first leaf with two pairs of leaflets and a complex tendril; III: 32 DPI when the floral bud is enclosed; IV: 42 DPI at the first open flower; V: 56 DPI when the pod is filled with green seeds; and VI: 90-110 DPI at the dry harvest stage. The values, which are not significantly different from each other (p ≤ 0.05) are marked with the same letter. Bars represent standard errors. (A) Fv∕Fm, the maximum PSII photochemical efficiency in the darkness-adapted state, Y(II), effective quantum yield of photochemical energy conversion in PSII; (B) qP, coefficient of photochemical quenching of chlorophyll fluorescence, qN, coefficient of non-photochemical quenching of chlorophyll fluorescence. contr, control plants, AM, plants inoculated with Rhizophagus irregularis. [file peerj-07-7495-s002.png]

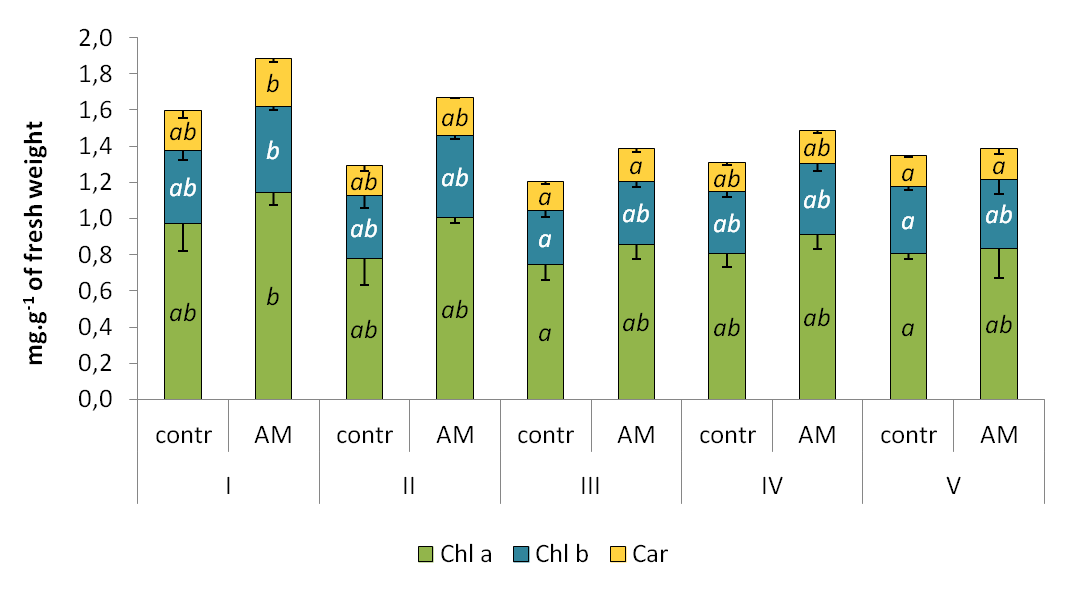

Supplement: Figure S3 — The values for each parameter, which are not significantly different from each other (p ≤ 0.05) are marked with the same letter. Bars represent standard errors. contr, control plants, AM, plants inoculated with R. irregularis. [file peerj-07-7495-s003.png]

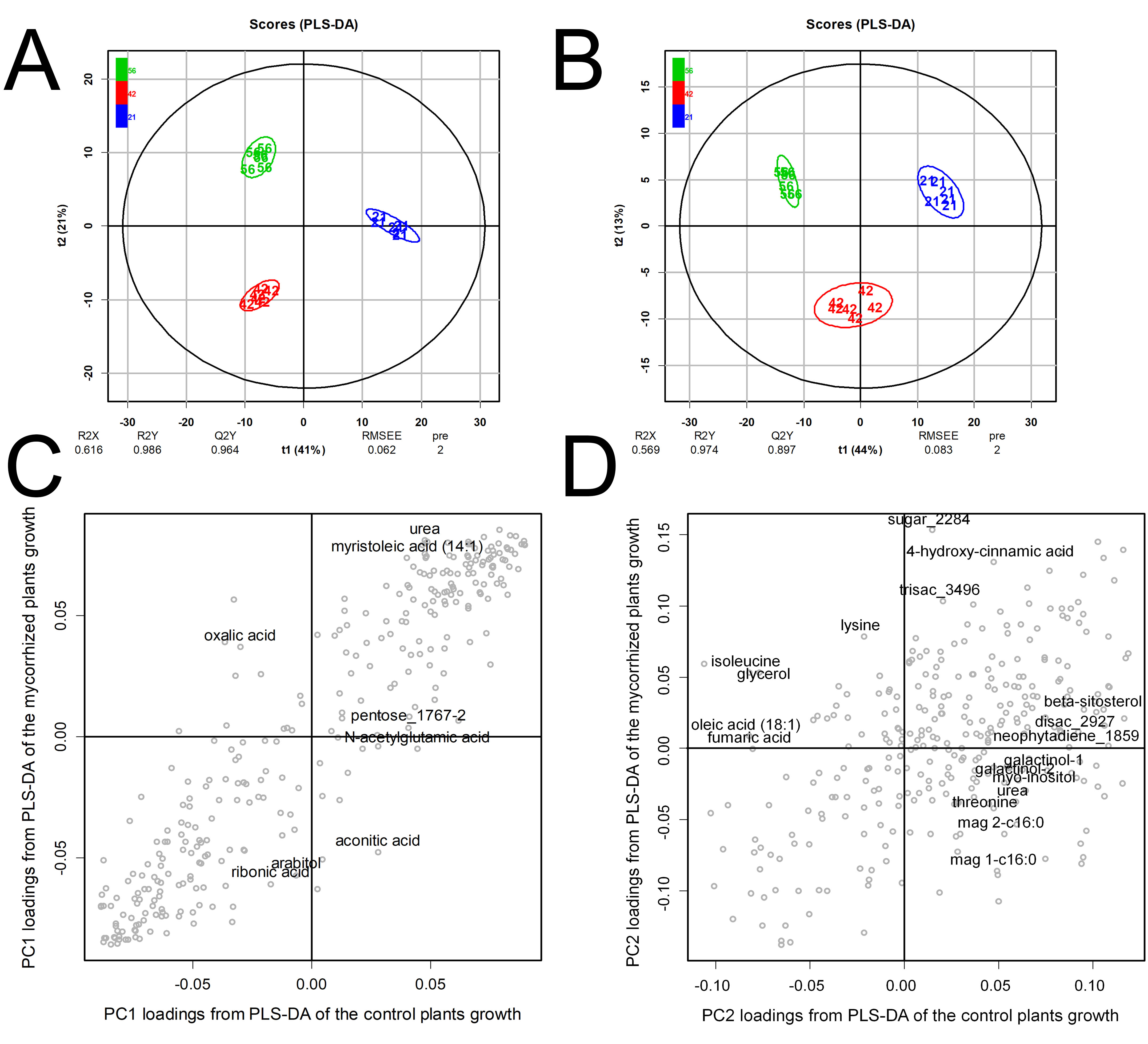

Supplement: Figure S4 — PLS-DA score plots with model parameters for control plants (A) and plants inoculated with R. irregularis (B). Scatter plots in the spaces of the PLS-DA loadings of: (C) PC1, (D) PC2. [file peerj-07-7495-s004.png]

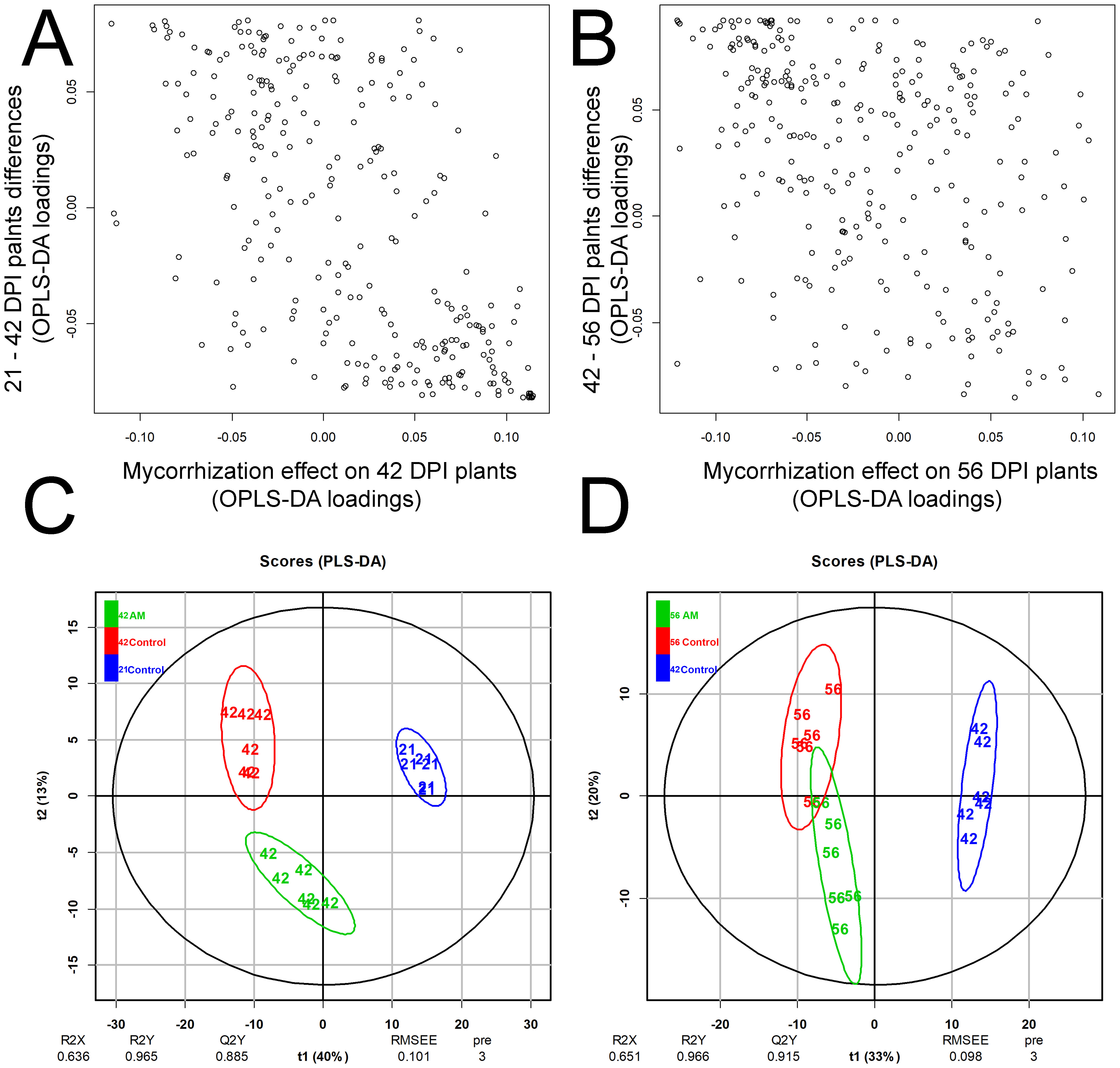

Supplement: Figure S5 — (A, B) Scatter plot in the spaces of the loadings of the predictive components from two OPLS-DA models: (A) First, for comparing 21 and 42 DPI plants; second, for control and inoculated (AM) 42 DPI plants, (B) First, for comparing 42 and 56 DPI plants; second, for control and AM 56 DPI plants. PLS-DA score plots with model parameters for the (C) 42 DPI control and AM and 21 DPI control, (D) 56 DPI control and AM and 42 DPI control. [file peerj-07-7495-s005.png]

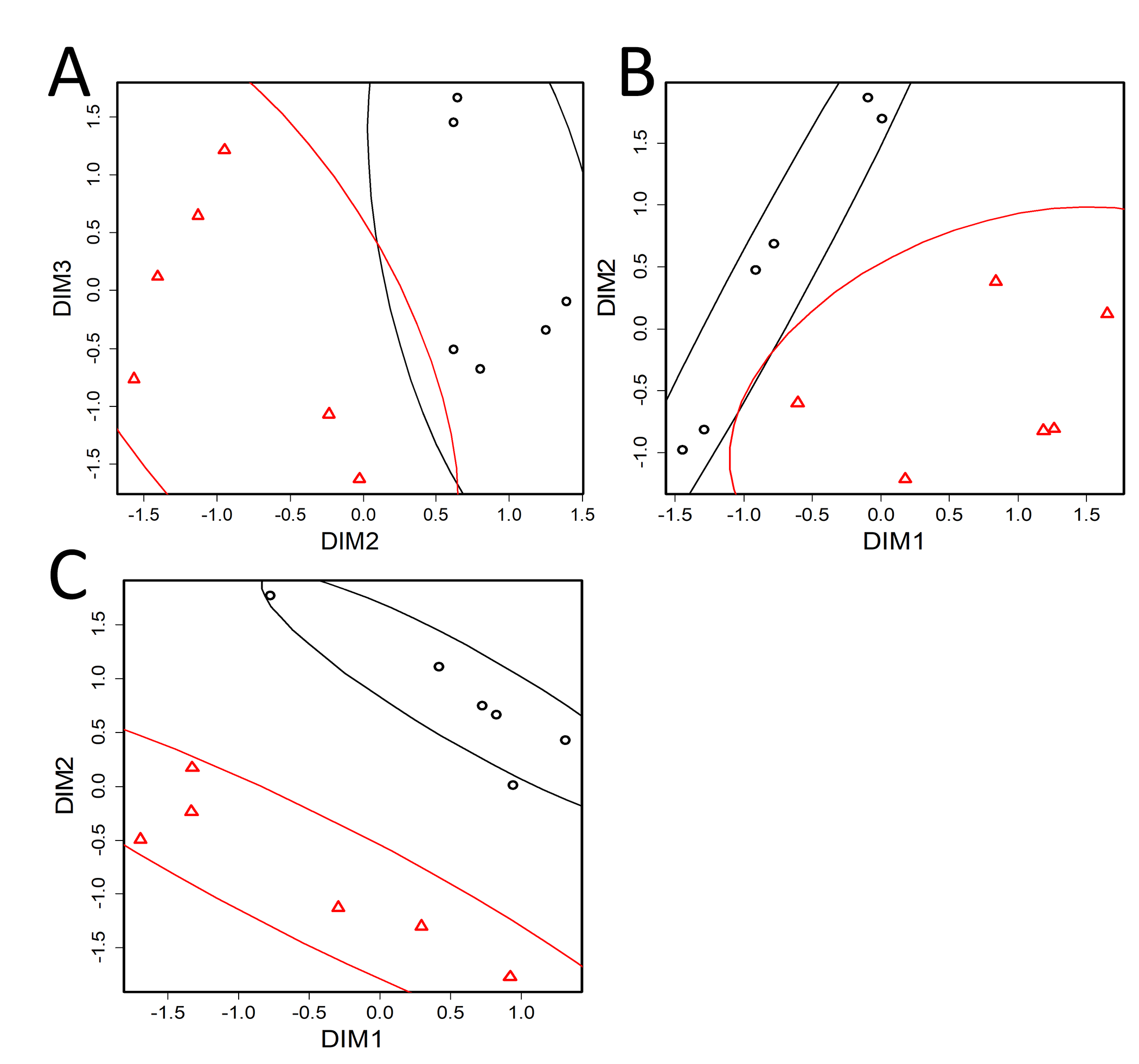

Supplement: Figure S6 — (A) 21 DAI (stage II), k = 10; (B) 42 DAI (stage IV), k = 6; (C) 56 DAI (stage V), k = 10. Black –control, red –plants inoculated with R. irregularis, ellipses –90% CI, DIM –dimension. [file peerj-07-7495-s006.png]

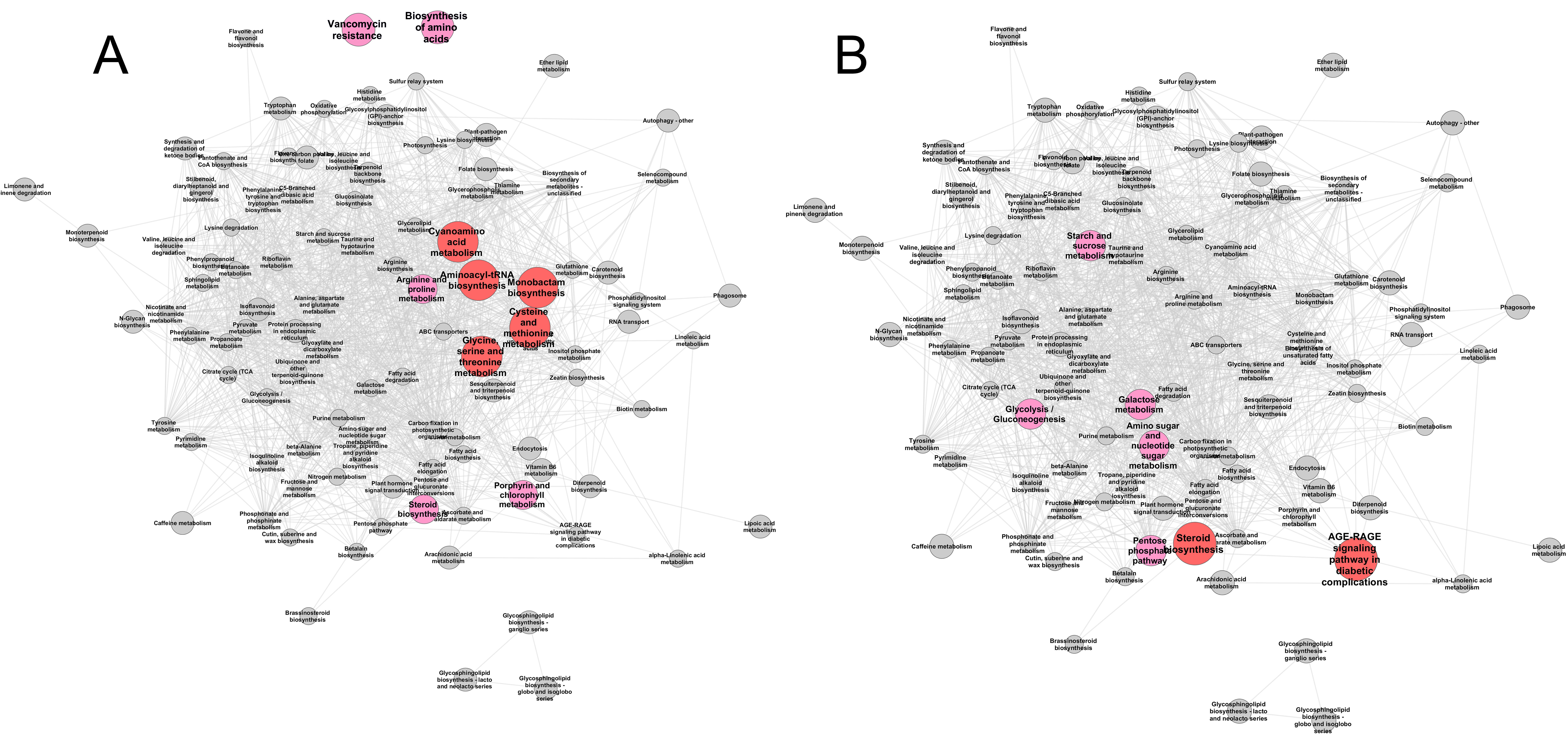

Supplement: Figure S7 — Pathway network based on KEGG database using Medicago truncatula as a reference species. Nodes (pathways) share common edge if they share metabolites. Graph was built in the Cytoscape environment using Prefuse Layout, where lengths of edges reflect the number of metabolites shared between pathways. The bigger red nodes correspond to p < 0.05, smaller pink nodes to p < 0.1, grey nodes to pathways sharing metabolites with significantly affected ones. (A) 42 DPI (stage IV); (B) 56 DPI (stage V). [file peerj-07-7495-s007.png]
